# A bacterial sensor taxonomy across earth ecosystems for machine learning applications

**DOI:** 10.1128/msystems.00026-23

**Published:** 2023-12-11

**Authors:** Helen Park, Marcin P. Joachimiak, Sean P. Jungbluth, Ziming Yang, William J. Riehl, R. Shane Canon, Adam P. Arkin, Paramvir S. Dehal

**Affiliations:** 1 Center for Synthetic and Systems Biology, School of Life Sciences, Tsinghua-Peking Center for Life Sciences, Tsinghua University, Beijing, China; 2 EPSRC/BBSRC Future Biomanufacturing Research Hub, EPSRC Synthetic Biology Research Centre SYNBIOCHEM Manchester Institute of Biotechnology and School of Chemistry, The University of Manchester, Manchester, United Kingdom; 3 Environmental Genomics and Systems Biology Division, Lawrence Berkeley National Laboratory, Berkeley, California, USA; 4 Computational Science Initiative, Brookhaven National Laboratory, Upton, New York, USA; 5 National Energy Research Scientific Computing Center, Lawrence Berkeley National Laboratory, Berkeley, California, USA; 6 Department of Bioengineering, University of California, Berkeley, California, USA; Boston College, Chestnut Hill, Massachusetts, USA

**Keywords:** metagenomics, machine learning, histidine kinase, sensory transduction processes, human microbiome, feature importance

## Abstract

Microbial communities have evolved to colonize all ecosystems of the planet, from the deep sea to the human gut. Microbes survive by sensing, responding, and adapting to immediate environmental cues. This process is driven by signal transduction proteins such as histidine kinases, which use their sensing domains to bind or otherwise detect environmental cues and “transduce” signals to adjust internal processes. We hypothesized that an ecosystem’s unique stimuli leave a sensor “fingerprint,” able to identify and shed insight on ecosystem conditions. To test this, we collected 20,712 publicly available metagenomes from *Host-associated*, *Environmental*, and *Engineered* ecosystems across the globe. We extracted and clustered the collection’s nearly 18M unique sensory domains into 113,712 similar groupings with MMseqs2. We built gradient-boosted decision tree machine learning models and found we could classify the ecosystem type (accuracy: 87%) and predict the levels of different physical parameters (R2 score: 83%) using the sensor cluster abundance as features. Feature importance enables identification of the most predictive sensors to differentiate between ecosystems which can lead to mechanistic interpretations if the sensor domains are well annotated. To demonstrate this, a machine learning model was trained to predict patient’s disease state and used to identify domains related to oxygen sensing present in a healthy gut but missing in patients with abnormal conditions. Moreover, since 98.7% of identified sensor domains are uncharacterized, importance ranking can be used to prioritize sensors to determine what ecosystem function they may be sensing. Furthermore, these new predictive sensors can function as targets for novel sensor engineering with applications in biotechnology, ecosystem maintenance, and medicine.

**IMPORTANCE:**

Microbes infect, colonize, and proliferate due to their ability to sense and respond quickly to their surroundings. In this research, we extract the sensory proteins from a diverse range of environmental, engineered, and host-associated metagenomes. We trained machine learning classifiers using sensors as features such that it is possible to predict the ecosystem for a metagenome from its sensor profile. We use the optimized model’s feature importance to identify the most impactful and predictive sensors in different environments. We next use the sensor profile from human gut metagenomes to classify their disease states and explore which sensors can explain differences between diseases. The sensors most predictive of environmental labels here, most of which correspond to uncharacterized proteins, are a useful starting point for the discovery of important environment signals and the development of possible diagnostic interventions.

## INTRODUCTION

Bacteria have evolved to colonize nearly every ecosystem on the planet. A key to their survival is the ability to sense and respond to diverse input (1). In prokaryotes, the sensor histidine kinases (HKs) are the primary signaling transduction system involved in environmental sensing (2, 3). Through their sensing domains, HKs can detect chemical and physical stimuli including pH, osmolarity, photons, toxins, proteins, and small molecules (4), and allow cells to react by deploying diverse cellular programs including cell division, biofilm formation, quorum sensing, antibiotic resistance, and virulence (5, 6). HKs are an extremely diverse class of proteins that, outside of a few well-studied archetypes, are still largely uncharacterized (7, 8). These proteins can be identified using their two conserved domains (Pfam: HATPase/PF02518, HisKA/PF00512) and can have zero, one or more sensory domains, which are the “eyes” used to detect fluctuations in their microenvironment (9). Currently, sensory domains are defined with Pfam domains; however, a single Pfam domain family can contain thousands of unique sensors that each respond to different environmental stimuli. For example, the well-characterized “PhoQ-like” family of HK proteins can sense shifts in pH, osmolarity, small antimicrobial peptides, and membrane proteins, suggesting that within this family there is enough sequence dissimilarity to create a diversity of sensor domain repertoire that detects different signals (8).

HK engineering is an effective target for biotechnology, medicine, and ecosystem monitoring. HK proteins have been engineered to be biosensors that accurately monitor stimuli (10), improve titer in bioindustry (5), and identify and trace environmental contamination (11). HKs in pathogenic bacteria regulate virulent secretion systems and antibiotic resistance and therefore are of interest for targeted pharmaceuticals (12). Targeting HKs is an attractive method to precisely block a microbial response without destroying the functionality of the surrounding healthy consortia, as is the typical result of antibiotic treatment (4, 13). For example, deletion of the HK PhoP in *Mycobacterium* and *Salmonella* results in microbes that are attenuated and immunogenic for virulence in animal models. Meanwhile, sensors have been engineered that allow bacteria to sense and infiltrate cancerous microenvironments and kill tumors (14).

It has been proposed that there is a direct relationship, up to a limit, between the number of sensory proteins and the complexity of the environment (9). For example, parasitic microbes that live in highly constrained and controlled environments tend to have small genomes with small numbers of sensors (9). Evolutionary events like domain shuffling are common in HK proteins, allowing for the rewiring of signaling networks without necessarily adding new sensing domains (15, 16). Prior research suggests a stable microbial state that is defined best by the community’s cumulative attributes; therefore, measurement of genomic indicators, such as the sensory profile, can more accurately represent a community than the more volatile taxonomic abundance (17, 18). Species phylogenetic information has been shown to be correlated with distinct ecosystems (19), to vary according to temporal shifts in their environment (20–22), and to predict functional microbial traits (23, 24). Functional protein domains can “cluster” microorganisms at the macro- (25) or micro-scale (26) by their environmental niche, suggesting that ecosystem prediction using genetic content is possible. For example, recently, a sensory protein index (SPI) metric was defined that was found to correlate loosely with *Escherichia coli* virulence and was used to identify particular pathogens that left distinct patterns in patients (27).

Recent efforts have been made to utilize “big data” microbial genomics and sequence features derived from metagenomic sampling over coarser taxonomic abundance to understand strain-level genetic variation in different ecosystems (28). For example, a study used clustering of public metagenomes to describe gene distribution in different biospheres and to elucidate gene families that are rare, abundant, or specific to certain habitats (29). Meanwhile, metagenomic analysis surveys have been used to identify new potential pathogens in certain urban areas from airborne samples (30) and to document the site specificity for microbial genetic signatures on the human body from skin samples (31). Recently, the first systematic prediction of ecosystem niche using prokaryotic genomic content was shown for different physical parameter gradients (ρ = 0.7–0.81) (32). The authors suggest this already strong association could be further improved after refining specific genes or functional families since variation in many protein families in biogeochemically distinct environments is minimal (33). Moreover, the sheer data size of genomic models using full metagenomic profiles limits analytical ability and speed.

In the following research, we aimed to see whether an environmental niche’s distinct HK sensor domain profile can serve as a basis for classification. To this end, we clustered sensory domains from 20,712 metagenomes covering a diverse set of ecosystems and taxa to create a sensor catalog. We then used machine learning modeling and feature importance to explore how the new sensor profile sheds insight into how microbes interact, sense, and respond to their environment.

## RESULTS

### HK sensor identification and clustering from environmentally diverse metagenomes

**Fig 1:**
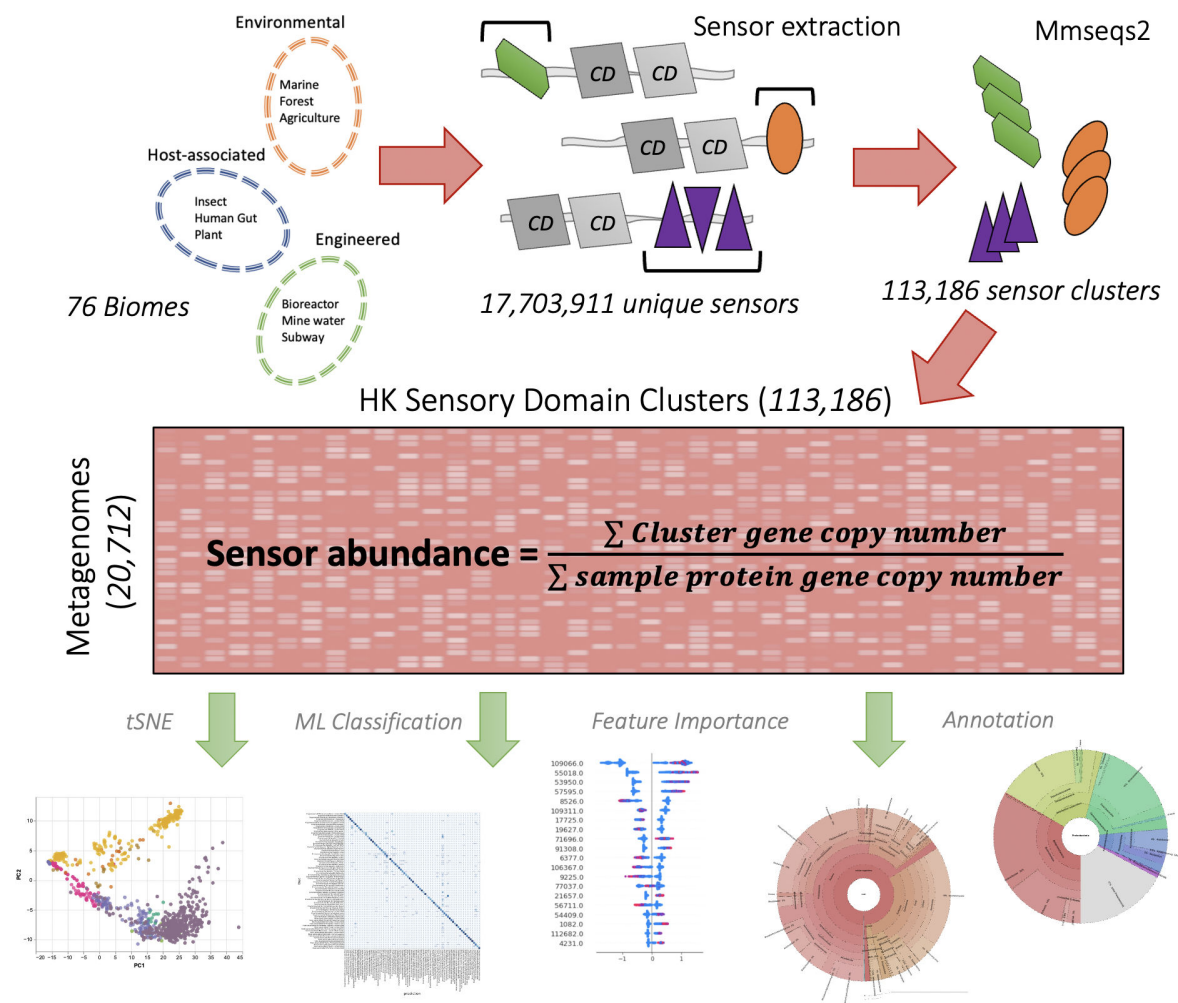
Overview of the data collection, sensory protein extraction, and curation of the matrix for machine learning. Experimental design. We first surveyed 20,712 metagenomes spanning over 75 ecosystems. We identified each metagenome’s HK proteins using two Pfam conserved domains (“CD”) and then extracted just the sensory domain from each protein using Pfam sensor domain annotations. We clustered 21,984,304 total proteins using MMseqs2 with 17,703,911 unique sensory domain sequences, leading to 113,186 clusters that may each respond to unique stimuli. We focused on clusters that were present in at least 100 metagenomes, dropping any rare features, leading to 14,990 final clusters for analysis for our ML models. These sequence clusters were used for machine learning, hierarchical clustering, and ecosystem sensor taxonomy. In the text, “full-length” proteins contain all sensory domains and conserved domains, while “sensor” references just the sensor domain of the HK protein. Both full-length proteins and isolated sensors used for clustering are protein-coding genes.

HK sensor profiles for individual metagenomes were constructed as a substrate for machine learning (ML) approaches to classify ecosystems and predict environmental parameters, discover new sensors, and facilitate a better understanding of the sensory repertoire in different ecosystems. We first identified HK proteins from 20,712 metagenomes spanning over 75 ecosystems, and extracted the amino-acid sequence for each HK’s sensory domain(s) (Fig. 1). Since HK domains are incredibly diverse even within classified domain families like Pfam, we opted to cluster domains at a higher degree of similarity than these standard methods to more precisely represent domains with common function. We hypothesized the use of HK domain clusters would result in a more precise sensory profile and would enhance the sensitivity of our models.

We next used MMseqs2 to group proteins by their sequence similarity. When performing MMseqs2, we first benchmarked with *Host-associated* ecosystems only and found that using the full-length HK amino-acid sequence led to 113,208 protein clusters compared to 33,825 for extracted sensor domains only (Table S2). We used only the individual extracted sensory domains for further analysis with the intent that each domain cluster could more meaningfully represent a common environmental stimulus without the influence of other HK protein domains (Fig. 1). The results from MMseqs2 were used to generate a sensor profile matrix where metagenomes were rows, sensor protein clusters were columns, and values are the fraction of genes in the sample represented by members of each protein cluster. Fractions are calculated by the count of genes assigned to the cluster divided by the total count of genes identified in the metagenome.

### Comparison of sensor clusters to Pfam domains

**Fig 2:**
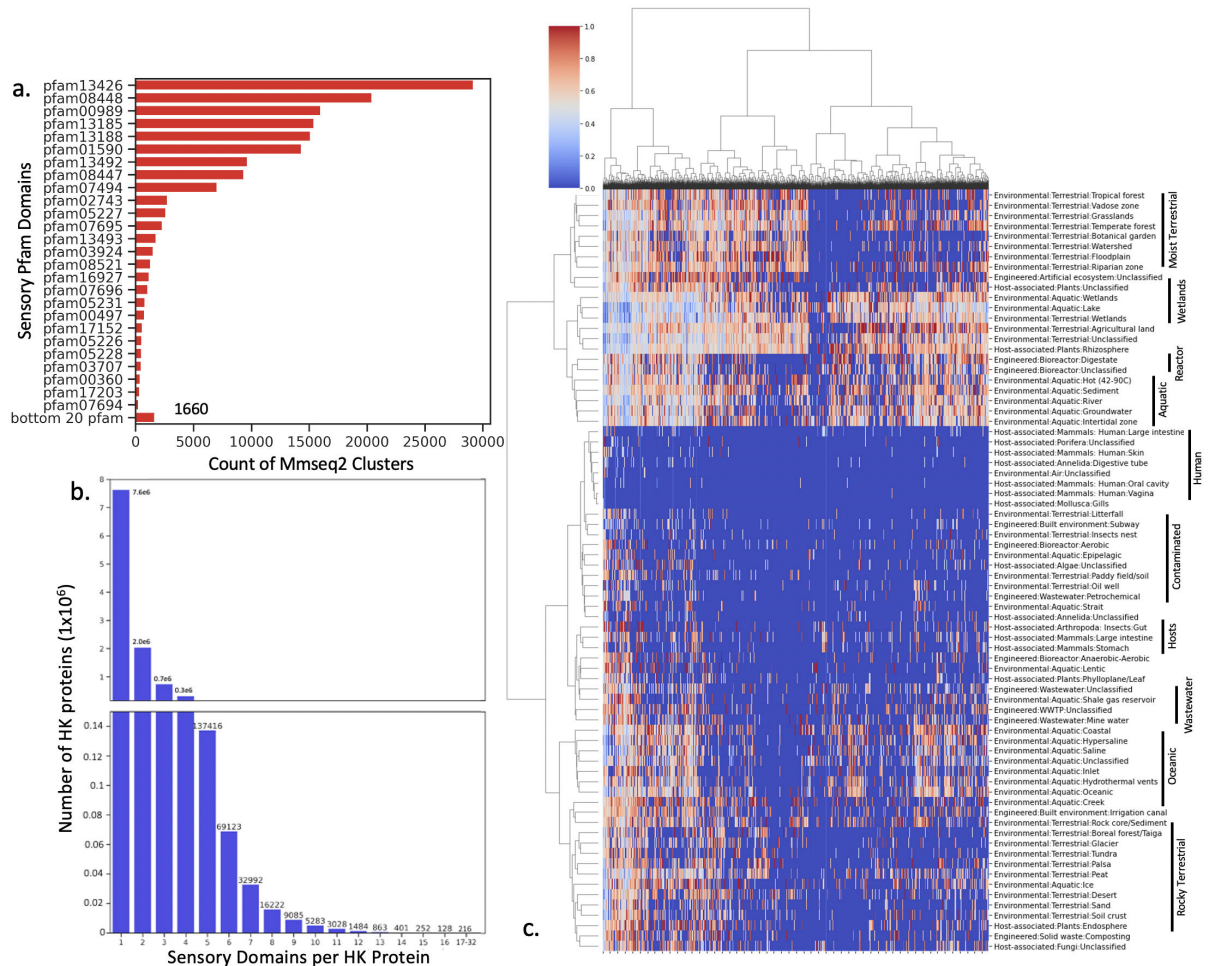
Similar sensory-domain clusters can predictably organize ecosystem types by increasing the complexity over Pfam identifiers. (**a**) The count of sensory clusters associated with each Pfam sensor domain. Many Pfams have a large number of clusters associated with them, for example, pfam13426 (PAS domain) is present in almost 30,000 clusters. The bottom 20 Pfam domains are summed in the final bar of the figure. (**b**) Shows the number of HK proteins that have a certain number of sensory domains. 43% of proteins have one sensory domain. The graph is split into two ranges to visualize all bars. (**c**) Heatmap (log2 scale) built using hierarchical clustering. Similar ecosystems group together in the heatmap, indicated with black bars, confirming that the information in the sensor profile matrix lends a predictable structure to the data set. For example, the *Environmental:Aquatic* metagenomics group, as do *Environmental:Terrestrial*, and *Host-Associated: Human,* etc. The Y-axis is ecosystems, and the X-axis is MMseqs2 sensory domain clusters, using the same abundance matrix used in ML training in later sections.

After clustering, we examined the relationship between the MMseqs2 sensor clusters and Pfam domains to determine whether the increased resolution due to finer groups would lead to higher precision. We found that most Pfam domains are associated with thousands of clusters, suggesting one Pfam contains proteins reacting to a huge variety of environmental stimuli (Fig. 2a). We next explored our HK sensory domains more closely and found the 11.5 × 10^6^ full-length histidine kinase proteins, most (7.6 × 10^6^) contained only one annotated sensory domain, although some contain up to 32 distinct sensory domains (Fig. 2b). Upon further investigation, the latter HKs consist of many small repeating transmembrane proteins. Finally, we used hierarchical clustering to better visualize the distribution of clusters across our ecosystems in a heatmap and tree enrichment diagram (Fig. 2c; Fig. S1). We found predictable groupings of similar ecosystems using these techniques, indicating there is structure to the cluster matrix that is interpretable on the ecosystem level. 98.7% of the sensor domain clusters have unknown functions, and only a handful have been extensively characterized, creating a valuable new catalog for both ecosystem and sensor exploration.

### Landscape of ecosystem diversity using MMseqs2 domain clusters

**Fig 3:**
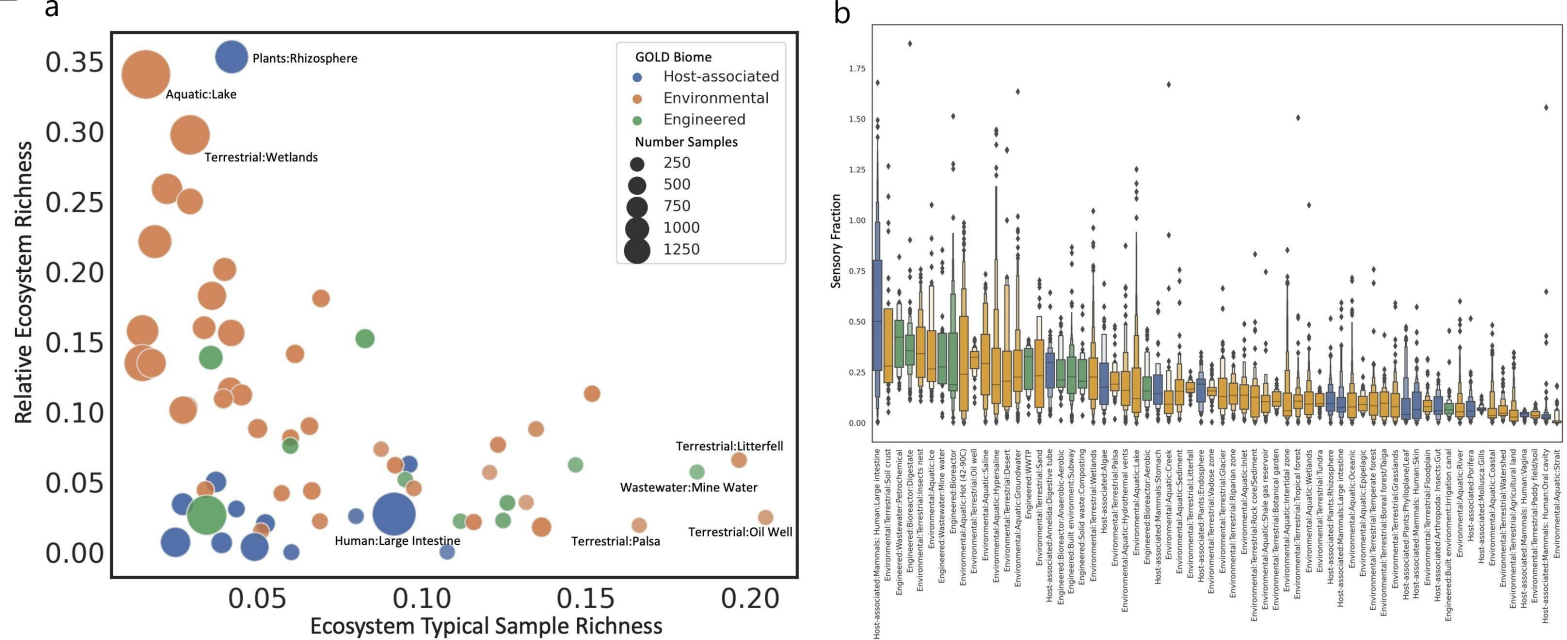
Relative Ecosystem Richness, Ecosystem Typical Sample Richness, and Sensor Fraction using Mmseqs2 clusters (**a**) Scatter plot of the ecosystem sensor Relative Ecosystem Richness (the proportion of total gene cluster diversity found within that ecosystem) and Ecosystem Typical Sample Richness (the proportion of the ecosystem’s gene cluster diversity typically found in a single sample). The size of each point is proportional to the number of samples taken from that ecosystem, indicating the extent of sampling. Generally, host-associated ecosystems display lower Relative Ecosystem Richness, with the exception of *Plant:*Rhizosphere; however, this is more similar to environmental ecosystems as it represents the interface between roots and soil rather than ecosystems contained within a host. More details can be found in Fig. S2. (**b**) Sensory fraction, the fraction of all proteins in a metagenome that are sensors. The fraction is shown as a percentage in the figure. Broadly speaking, we observe individual samples can approach a ceiling, indicating that scaling sensing by individual sensors has some natural limit. Microbial populations may only energetically commit to make a certain fraction of sensory proteins from their protein pool. There is also a large range seen between ecosystems from a mean of 0.05 (*Human:Oral Cavity*) to 0.5 (*Human:Large Intestine*).

Prior research suggests HK sensor diversity scales with ecosystem complexity and that constant environments require a minimal number of sensing proteins (9). We explored the diversity of sensor clusters across the ecosystems in our data set using two calculations. First, Relative Ecosystem Richness (RER) is the fraction of all unique clusters found in an ecosystem. Second, Ecosystem Typical Sample Richness (ESTR) is the fraction of an ecosystem’s clusters found in the average sample from that ecosystem. Together, these two measurements show the sensor space covered by an ecosystem and how much average samples within an ecosystem cover its sensor space (Fig. 3a). By the nature of the calculations, these two measures are expected to be anticorrelated because a sample is less likely to cover all clusters if it is from an ecosystem with a large number of unique clusters. However, we observe that the ecosystems with high RER and low ETSR are more spatially and environmentally heterogeneous, which may indicate their class labeling is too broad and are labeling physically distinct biome types. The degree of spatiotemporal variation in a single geographic region may also drive defined microbial successions and therefore require distinct functional profiles (34). This can lead to high RER due to higher diversity required and lower ESTR because different samples are caught at different moments in space time. Finally, some of these ecosystems have been measured hundreds of times and others only a few. In Fig. 3, the ecosystems with the highest RER and lowest ESTR have labels most likely to be affected by all three of these effects, namely label specificity, degree of spatiotemporal variation, and ecosystem observation count. For example, the label “Plant:Rhizosphere” might group together microbial communities that are proximal and distal to plant roots from many cropping conditions. Indeed, this class contains 981 metagenomes, from 179 different geographic locations, and some studies only contain a single sample. This reduces the chance of highly conserved features for this type of biome and may make it difficult to unambiguously predict a sample’s class from its profiles because the variance uncertainty is so large.

We also calculated the sensor percentage, the percentage of all proteins in a metagenome that are sensors (Fig. 3b). *Human:Large Intestine* has the highest percent of sensory proteins, but a low cluster richness, and also has the highest number of clusters that are found rarely in other ecosystems (Fig. S1).

### A gradient boosting on decision tree regression model can predict ecosystem physical parameters using sensory profiles

Our central hypothesis is that sensory domains are predictive of ecosystem class. The definition of class, however, is imperfect as real ecosystems are not cleanly separable by discrete ontological labels. For example, where two types of environments “interface” such as water and land at the littoral zone there is a continuum of change from water to dry land. Furthermore, even within a seemingly homogeneous class like “Sediment,” there may be variation over parameters such as pH or temperature among others. Thus, we used ML classifiers or regressors to make broad predictions of class or precise predictions in parameter variation within a class, respectively. In both cases, we used the Shapley Additive exPlanations (SHAP) (35, 36) to rank our features most important predictors, corresponding to the specific sensor domain clusters.

As a first demonstration, we show that domain profiles can predict levels of continuous environmental parameters within an ecosystem class. That is, they can act as biosensors for these physical signals. We selected metagenomes from the *Aquatic:Marine* class as our input data for a CatBoost regression model which uses gradient boosting on decision trees. These samples were associated with physical variations in temperature, pH, salinity, chlorophyll, nitrogen, and oxygen concentration, each of which we trained to predict from the domain clusters from these metagenomes. After optimizing the model hyperparameters, we were able to achieve an R2 score of over 0.83 for parameters salinity, pH, and nitrogen concentration, and 82.7 for temperature (Fig. S3). Meanwhile, the R2 for chlorophyll only reached 60.8, perhaps because only one-third of the metagenomes had values for this parameter. Perhaps more significantly than the final R2, we isolated the top features (sensor domain clusters) that drive the model’s prediction for each physical parameter. We explored the temperature model because it had the broadest predictable range, but found the top 20 features were not annotated and therefore unknown (Fig. S3b). We did find the HK taxonomic classification indicated the proteins are found in marine bacteria, for example, ***cluster*:*49561*** was annotated to be an unknown HK found in SAR324, a marine organism documented to thrive in hydrothermal plumes. For this sensor, high feature importance corresponds to a higher temperature (Fig. S3d). However, without adequate annotation and indeed experimental validation, further interpretation of the regression models is speculative.

### A gradient-boosting decision tree classification model can accurately classify ecosystems using sensory profiles

Although the regression models indicate the sensory profile can predict characteristics with a reasonable R2 score, few ecological subsets of metagenomes in our data set have enough members labeled with a quantitative physical parameter such that regression is possible. Moreover, we noticed that the *Marine*, and indeed *Environmental* ecosystems overall, tended to have mostly unstudied sensors. Therefore, we proceeded to build a CatBoost classifier using all metagenomes from all ecosystems, with the goal for the model to predict the ecosystem ontological label for a metagenome from the sensory profiles. We used hyperparameter tuning, grid search, and feature selection (**Methods**, Fig. S4) and reached an accuracy of 0.87 on a non-test set with a train/test/validation set split of 70/20/10. We also compared the ML performance for the 42 Pfam domains and MMseqs2 clusters using an otherwise identical set of hyperparameters. We found that the model trained with the MMseqs2 clusters led to a noticeable improvement upon the model trained on Pfam domains [0.87/0.98 versus 0.78/0.98 (test/train set accuracy), respectively). This suggests that the protein clusters allow for more precise sensor protein characterization than Pfam and therefore more accuracy in ecosystem characterization. Finally, we found the final accuracy changed minimally (±0.02) with different hyperparameters, but that the composition of input data (as in, if a particular ecosystem class was removed and the model was retrained), impacted accuracy significantly.

The confusion matrix for classification can be used to diagnose which ecosystems are predicted well or poorly by the model and for those predicted poorly which ecosystem they are misassigned to. We found that most mispredicted ecosystems are in *Environmental* habitats, for example *Terrestrial:Soil crust* (33% predicted *Plants:Rhizosphere*), *Aquatic:Inlet* (29% predicted *Aquatic:Coastal*), and perhaps a bit concerning, *Wastewater:Petrochemical* (25% predicted *Aquatic:River)* (Fig. S4). We also noticed while testing the model that completes the exclusion of *Environmental* ecosystems still left 38% of the data set but lifted accuracy to 0.95, even before any additional grid search optimization. We reasoned that ecosystem labeling is not perfectly standardized and labels are often underdefined, especially in *Environmental* ecosystems. To test whether ecosystems are mispredicted because they have similar sensor profiles, we calculated the Spearman correlation for the above three cases (coefficient: 0.443, 0.516, and 0.350, respectively; *P*-value < 0.05). However, these are far from the most correlated ecosystems; a correlation matrix indicates 16 ecosystems pairs have a correlation of over 0.8 (Fig. S5) and the highest was 0.87 (*Terrestrial:Sand*, *Terrestrial:Soil Crust*), two ecosystems predicted correctly by the model. In the end, 66.5% of ecosystems in our final model have an accuracy above 0.85, suggesting the sensor profile can accurately classify ecosystems.

#### Annotations of feature-important sensor clusters help interpret physical differences among ecosystems

The top two most predictive features in our ecosystem label classifier are ***cluster*:*109066*** and ***cluster*:*109311*** (Fig. S6). Annotations for both are for a putative oxygen sensor analogous to *fixL/dosP* and were discriminatory for the prediction of all classes. The presence, absence, and abundance of oxygen-sensing proteins appear to be a key differentiator between ecosystems. We noticed, after annotation of the sensor domains, that certain sensors are incredibly widespread (i.e., *walK,* an HK essential for cell wall formation and cell division in most bacteria) while others are rare (i.e., *Mycobacterium tuberculosis*’ sensor *dosS*). Sensors like walk were found in nearly all metagenomes and appeared to be discriminatory for all classes in the model.

### Important domain clusters identify sensors for oxygen and disease status in human tissues

To examine in detail the power and limitations of sensor profiles in interpreting the differences among ecosystems, we chose to more deeply explore the *Host-associated* ecosystems. We selected these ecosystems because we expected the signal annotated from the most predictive cluster(s) in the model, oxygen, to be a critical differentiating factor. We used the top important domain features from our classifier to determine whether they cluster different tissue classes.

**Fig 4:**
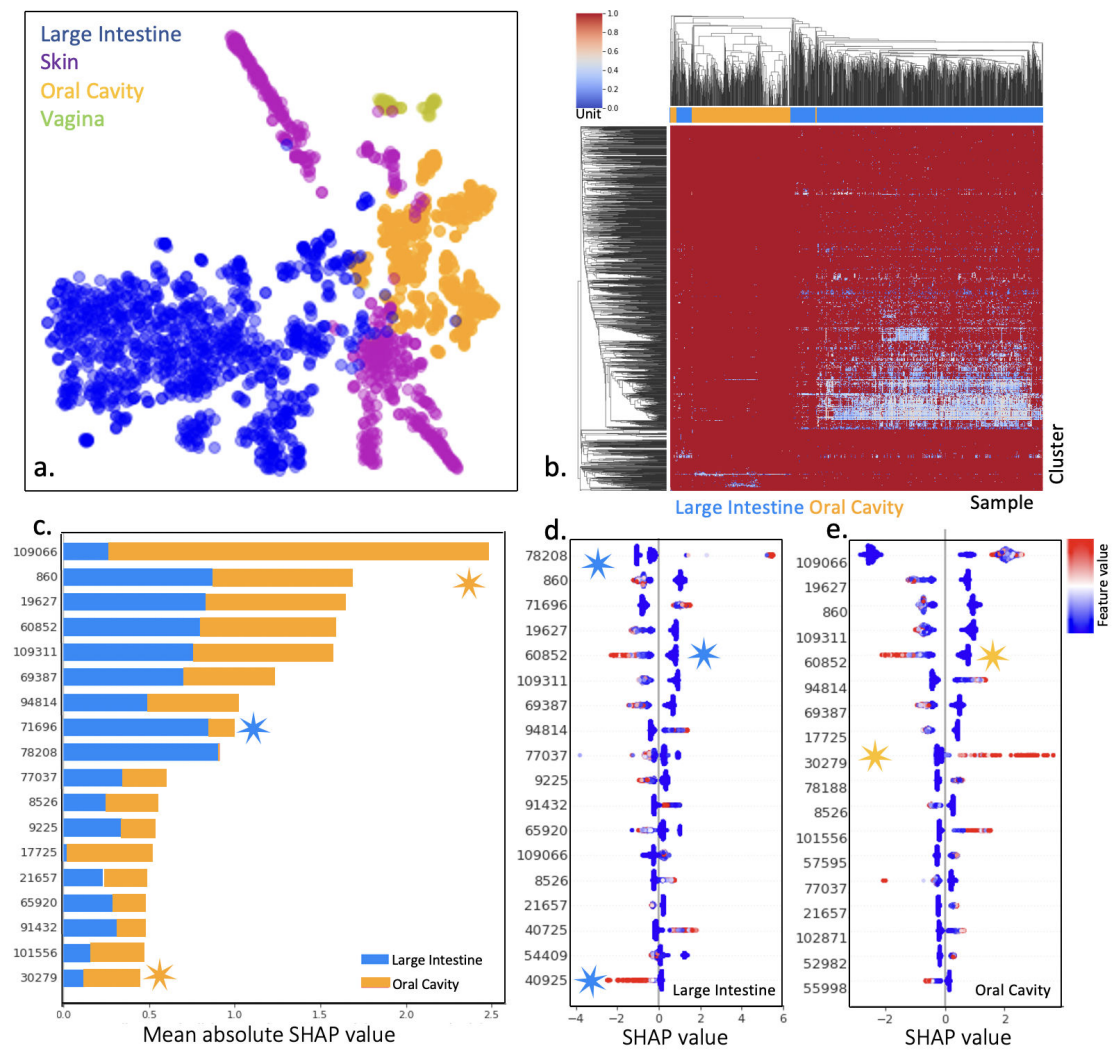
Feature importance of sensory clusters explain differences between gut and mouth**. (a**) t-SNE plot for all metagenomes in *Host-associated:Human* ecosystems using the sensor profile, colored by tissue. (**b**) Hierarchical clustering and heatmap for large intestine and oral cavity ecosystems reveal clear groupings and a few discriminatory features in *Large Intestine*. (**c–e**) Feature importance for *Oral Cavity* and *Large Intestine*. For feature importance, the rank for a feature indicates how impactful that feature is in the ecosystem classification. This method sorts the features by the sum of importance values across all metagenomes to understand the impact a feature has on the output class. The bar chart (**c**) sums the absolute value for both ecosystems, while the scatter plots are for *Large Intestine* (**d**) and *Oral Cavity* (**e**). In the SHAP force plots, the Y-axis is the feature’s importance rank, the X-axis is the feature importance value, and each dot represents one metagenome. A positive importance value (X-axis) will lead the model to select for the class, and a negative value to select against the class; the color spectrum represents the value of the feature compared to other classes (red: high, blue: low abundance).

A t-SNE plot derived from a matrix of these domain clusters from relevant metagenomes shows clear separable groupings of the metagenomes from different tissue classes based on these domain cluster profiles. An initial t-SNE indicated that the sensory matrix can create clear groupings along the human tissues (*Vagina, Skin, Oral*, and *Intestine*) (Fig. 4a). We selected *Large Intestine* and *Oral* ecosystems to focus on because they represent, respectively, a strong gradient from low to high oxygen, and also incidentally the full range of high to low fractions of sensory proteins per metagenome (Fig. 2b). A heatmap indicates certain sensor clusters are particularly enriched in the *Intestine* (Fig. 4b). Figure 4c indicates the most differentiating feature from the original model is ***cluster*:*109066***, which is annotated as a *fixL* putative oxygen sensor.

### Oxygen sensors can distinguish gut and oral metagenomes

It is logical that oxygen would be a differentiator between the gut, which typically consists of facultative and obligate anaerobes and the aerobic mouth. Indeed, high values of ***cluster*:*109066*** indicate the *Oral* ecosystem (Fig. 4c and e). However, the feature that is most predictive of a gut versus oral community is ***cluster:78202*** which represents an uncharacterized domain cluster that is largely found in the abundant “healthy” gut bacterium *Bifidobacterium longum* (37). This bacterium is dominant in the more aerobic infant gut but remains a significant fraction in anaerobic adult guts (38). Indeed, most top features whose over-abundance is predictive of the *Large Intestine* ecosystem are unknown HK sensors found in commensal gut bacteria, for example, cluster ***cluster*:*40725*** is an unknown HK found mostly in the abundant gut microbe *Lachnospiraceae,* another obligate anaerobe (39).

### A biofilm-associated domain is predictive of health-associated bacteria in the mouth, and other human-associated ecosystems

In the *Oral* ecosystem, one of the most important features whose over-abundance is predictive of the oral environment was ***cluster*:*30279***. These are predominantly annotated as sensor VicK, an HK is known to play a role in cavity formation. BLASTp query confirmed that one sequence within this cluster is an exact match for VicK in the cavity-causing bacteria *Streptococcus mutans*. We found members of ***cluster*:*30279*** in a few other ecosystems (*Human:Skin, Human:Large Intestine*, and *Subway*); however, annotations for the full HK protein in these ecosystems are for the *walK* family (Fig. S7), a common biofilm-inducing HK analogous to VicK in *S. mutans* (40). Indeed, taxonomic enrichment for each ecosystem revealed 98% *Staphylococcus* (majority *S. epidermidis* and *S. haemolyticus)* in non-oral human and non-host ecosystems, and 61% *S. mutans* in *Oral* ecosystems (Fig. S7). This implies that this biofilm-associated HK plays an important differentiated role in the microbes found in different human environments.

### Indicators for disease state and conditions in the *Large Intestine* ecosystem

In our analysis, the *Human:Large Intestine* had a relatively low number of unique sensor clusters despite having the highest number of rare clusters, and the highest sensor fraction compared to other ecosystems (Fig. 3b). Since prior research suggests disruptions to a diversified gut ecosystem can promote or reflect a disease state (41), we tested the functionality of our catalog by building a classifier within the *Large Intestine* to predict a patient’s disease state using the sensor profile. Specifically, we hoped to determine whether non-normal patients would have divergent sensor profiles in our data set.

**Fig 5:**
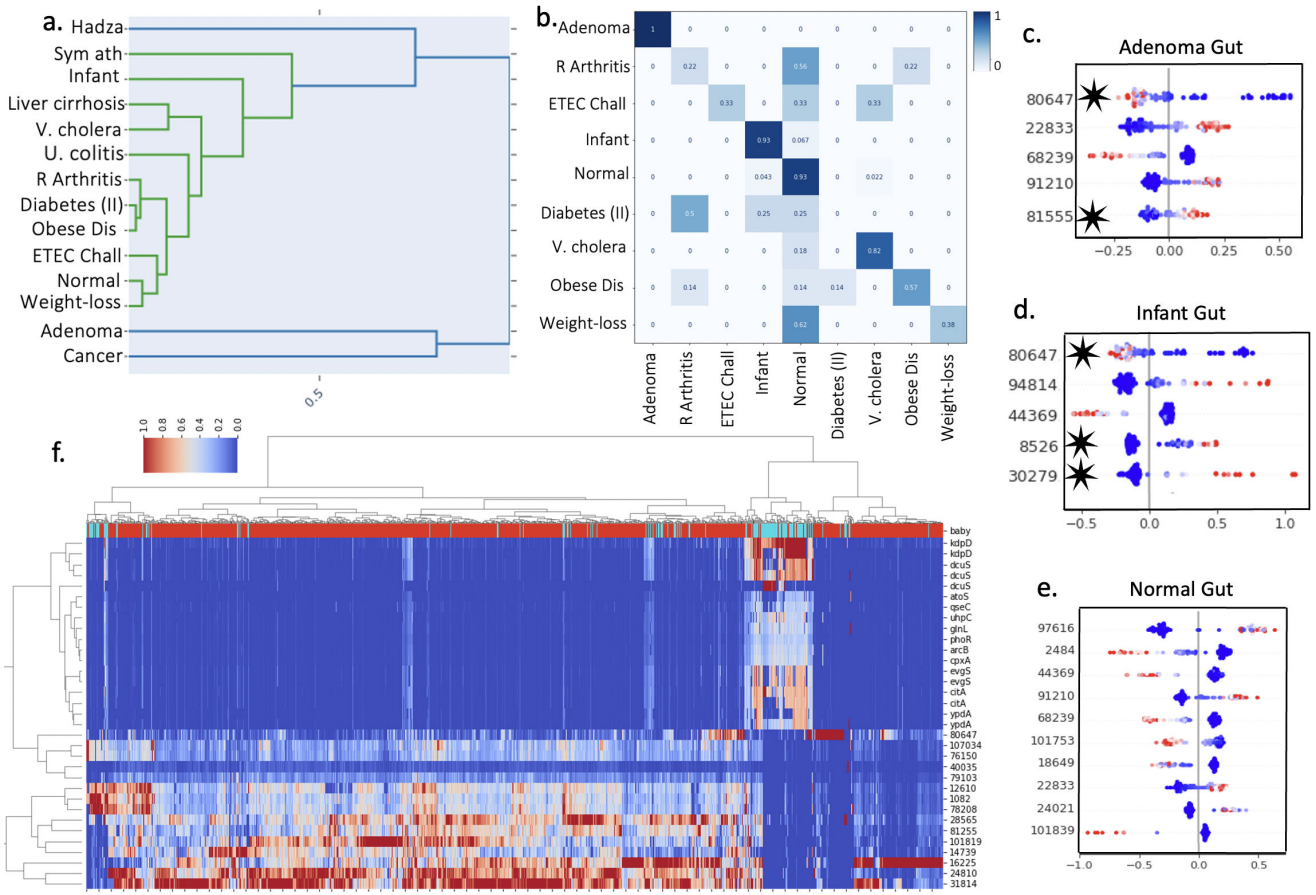
Disease classes can be predicted from the sensor profile to make meaningful insights. (**a**) Hierarchical clustering across normal and disease conditions in *Human:Large Intestine* metagenomes using sensory profiles. This dendrogram based on the similarity of the HK sensor profiles shows that samples labeled as Ademona (non-cancerous tumors) and Cancer form an outlying group and thus represent the most different samples and ecosystems in this set. Conversely, certain other ecosystems exhibited higher similarity such as normal and weight loss and type II diabetes and obesity (online supplementary file 7c). (**b**) A confusion matrix from the CatBoost classifier, with disease class as predicted label class. (**c–e**) Top features (clusters of sensory domains) in the Adenoma, Infant, and Normal gut classes. Features discussed in the text are indicated with red stars. (**f**) Cluster heatmap with X-axis as all gut metagenomes, Y-axis as all clusters that are correlated and anticorrelated to the sensor QseC. Acronyms in (**a, b**) correspond to disease classes: R Arthritis: Dysbiosis in Rheumatoid Arthritis; ETEC Chall: ETEC H10407 challenge study; Diabetes (II): type II diabetes; V. cholera: V. cholera challenge study; Obese Dis: microbial dysbiosis in young adults with obesity; Weight loss: obese patients following a weight-loss intervention; Hadza: Hadza hunter-gatherer gut microbiota; U. colitis: ulcerative colitis fecal transplant; Sym ath: symptomatic atherosclerosis; Dendrogram: Distance between sensor profiles.

Hierarchical clustering revealed the *Large Intestine* ecosystem can be meaningfully subdivided into different conditions (Fig. 5a). Visual inspection of the dendrogram suggests that there are strong clusters in the HK features that distinguish disease states. We used Spearman correlation and found that “adenoma” and “cancer” metagenomes are structurally uncorrelated (<0.02 coefficient) from other condition classes, while “Type 2 diabetes,” “young adults with obesity & microbial dysbiosis,” and “dysbiosis in Rheumatoid arthritis” are correlated (>0.89 coefficient for each). We next trained a CatBoost classifier using conditions as labels, to discern whether the sensor profiles have predictive power. After a grid search for hyperparameter tuning, we achieved a test/train accuracy of 0.79/1.0. The confusion matrix shows that some classes were consistently predicted correctly (Adenoma, Infant) and those confused were not surprising given the conditions we had access to in the data set (“Obese patients following weight loss intervention” mispredicted as “normal,” etc.) (Fig. 5b).

Encouraged by these results, we next looked at feature importance and initially noticed multiple high-rank features have similar patterns between classes, indicating those sensors were stable across patients. However, certain clusters diverged from normal. For example, we found the absence of ***cluster*:*80647*** is predictive for infants, adenoma patients, and diabetes patients, but conversely, the presence is of high feature importance in normal subjects. BLASTp indicates this unknown HK sensory domain is from *Faecalibacterium prausnitzii*, a normal commensal bacteria in the gut (42). Indeed, many top clusters in disease classes revealed key healthy commensal bacteria were low in certain diseases. For example, ***cluster*:*16225*** and ***cluster*:*24801*** were unknown HK sensors in the *Phocaeicola vulgatus* genome, while ***cluster*:*79103*, *cluster*:*1082*** corresponded to *Bacteroides uniformis*. Low abundance values for these clusters were indicative of adenoma patients, while normal patients had positive SHAP values indicating higher relative sensor abundance.

Meanwhile, we found ***cluster*:*81555*** was a positive disease indicator in adenoma, rheumatoid arthritis, and young obese adults with dysbiosis, but was low in normal patients. BLASTp indicates this unknown HK is found in *Parabacteroides distasonis*, an opportunistic pathogen that tends to bury in the gut tissue lesions in Crohn’s disease patients (43). Another promising positive indicator was the VicK***cluster*****:*30279*** identified previously as abundant in infant’s gut and an important feature for prediction. ***Cluster*:*8526*** also had high feature importance in Infants and was annotated as an oxygen-sensing domain (NitB) in *Escherichia coli*.

The cluster containing the QseC domain also stood out in the metagenome collection, since this HK is a recognized identifier for enterohemorrhagic *E. coli* (EHEC). Interested in investigating how our sensory profile could explore QseC in the *Large Intestine* samples, we performed dimensionality reduction using tSNE for the sensor profiles and found 12 sensory protein clusters grouped with QseC (Fig. S8). Multiple of these 12 sensory profiles were of high feature importance when differentiating classes within the *Large Intestine* ecosystem, although they were not the most important features (only 2 in the top 10). We then used a heatmap to determine whether QseC and clustered sensor profiles showed meaningful sub-structure in our data set. The heatmap dendrograms revealed clear clustering of the infant class, indicating that QseC is more elevated in infant metagenomes than the rest of the data set (Fig. 5F). Moreover, upon literature search, we found each of these HKs had annotations associated with human disease states (Fig. S8). Although together these results are not quantitative, feature importance and dimensionality reduction can bring focus to new sensors for further study in an otherwise expansive and unannotated data set especially for those with a targeted research question or ecosystem.

## DISCUSSION

Extensive research has used metagenomic functional and genomic diversity to explore ecosystem conditions, but few systematic models have been presented for ecosystem classification. Efforts have been made to use functional profiles to predict physical parameter gradients in an ecosystem (44, 45). Tools have been created to predict the flow of organisms or functions from one environment to another, for example, the package SourceTracker is a Bayesian approach that can predict environmental contamination from metagenomic profiles (46). However, SourceTracker is most useful when there is a known physical relationship for dispersal among the systems being studied. In our method, we are not considering such relationships although there is evidence for dispersal in environments which are confused in our predictions. However, to utilize the SourceTracker tool, community sources and sinks must be considered and standardized, and for our multi-environment data set, there is not an obvious or consistent way to define sources and sinks. A second typically disregarded but important limitation for predictions that use the full taxonomic or functional profile is the sheer size of data sets. Our method does not require taxonomic prediction, and the data set used for analysis consists of a greatly reduced matrix with counts of clustered protein families. Finally, we believe a key benefit of our developed method is in the selection of HK sensory domains as the sole functional group for model training. This selection creates the opportunity to form linkages between niches and environmental stimuli, adding explorable interpretability to our results.

In this research, we have found that sensor-abundance profiles can lead to accurate ecosystem classification and prediction of physical parameters. We found domain clusters are a powerful predictor of classes and physical parameters in diverse environments and can be used to identify the critical signals that adapt microbial communities to different niches. Well-annotated sensor clusters can identify possibly critical environmental resources or stressors that most distinguish an ecosystem and its state from others. If a domain cluster is localized phylogenetically, it can indicate critical taxa for the exploitation of these environmental stressors. We also found the sensor profile was precise enough to cluster and identify rare HKs. For example, we found a cluster annotated to be *Mycobacterium tuberculosis* (*Mtb*) sensor *dosS*. The protein *dosRST* is found only in *Mtb* and therefore has been of interest for drug development, as its inhibition can shorten tuberculosis therapy and kill persistent bacterial cells (47). We found the cluster was in only one study in our data set, for the mummified remains of an 18th-century tuberculosis patient (48). This indicates that our sensor clusters are precise enough to retrieve information for individual species and could be used to identify new HK targets.

We found in the human oral and gut ecosystems results of our models showed consistency with other data from the field. For example, it has been well documented that initial infant gut colonizers are aerobic (49), and indeed, we found high feature importance for the VicK (***cluster*:*30279***) and *nitB* oxygen sensor (***cluster*:*8526***), supporting existing evidence for an aerobic infant gut as microbes traverse from mouth to intestine during initial colonization (50, 51). A distinguishing feature of an infant from a mature gut is that the former is aerobic and the latter anaerobic. We find that specific oxygen sensors known to be operational in the aerobic oral environments are differentially found in the infant gut but not the adult gut implying the ability of the infant oral microbiome to traverse the intestinal tract. We also found VicK as an important feature in the *Oral* ecosystem, which is notable because this HK is known to incite cavities through initiating extracellular polymeric substances (EPS) synthesis and plaque biofilm (52, 53). Prior protein engineering efforts have created VicK mutants deficient in biofilm formation that limit cavities (54). Interestingly, we found (***cluster*:*30279***) in non-*Oral* ecosystems as well; however, these sequences taxonomy corresponded to *Staphylococcus* WalK, indicating both VicK and this WalK variant may react to similar, currently unknown, stimuli. Mutating VicK could be one preventative method designed for *Staphylococcus* to alleviate cavities (12).

We find in this research that the *Human:Large Intestine* ecosystem has the highest number of rare clusters, and the highest fraction of sensory proteins, but has a relatively low sensor diversity compared to other ecosystems. The CatBoost classifier for disease state revealed many top clusters could differentiate between normal and disease state classes. Our model identified ***cluster:80647***, a domain of unknown function, as high in normal and low diseased gut populations. This specific cluster seemed to be limited to the presence of *F. prausnitzii* which in other studies have been shown to be depressed in disease states including irritable bowel syndrome (IBS), cancer, and obesity (55). Our model also identified ***cluster:8526*** as an important feature for distinguishing human tissues. This domain is oxygen sensing and is in high abundance in the oral and infant microbiomes which are aerobic but depressed in adult gut samples which are anaerobic. We also found domains in bacteria known to play key functional roles in diseases in specific tissues, like *vicK* and QseC. QseC, an HK that causes flagella production and increased motility, was found to be elevated in infant classes. EHEC are serious food and waterborne pathogens associated with numerous outbreaks worldwide, and they rely on HK QseC to swim close to the mucosal epithelium and colonize (56). There are drugs in development that inhibit this HK’s sensory domain *via* steric hindrance to stymie virulence (57). Our data also suggest that the infant and adult gut ecosystems are distinct in taxonomic composition, with QseC and correlated HK sensors in high abundance in infants but low in adults. Indeed, the gut is known to diversify into adulthood, tending to stabilize with “healthy” colonizers unless there is a perturbation such as antibiotic therapy. Such an event can regress the microbiome back to an infantile state of meager microbial diversity, increasing the chances of pathogenic penetration and impacting overall health (58, 59). Taken together, our findings indicate the infant’s gut is fairly aerobic and susceptible to pathogenic bacteria. The consistency of our model-derived feature importance and their mapping to taxa and functions observed to be important in the literature supports the utility of this approach.

Once a sensor profile is defined in an ecosystem, it can be used to monitor deviations from a “normal” healthy state. Our CatBoost regression model results indicate the sensor profile can be used to predict physical parameters, and there is therefore great potential for HK research in *Environmental* ecosystems. Apart from their diagnostic potential, the sensors we predict as being abundant and discriminating for key environments are possible candidates both to target to remove unwanted microbes from those environments and as elements of engineered bacteria to enable designed responses to specific environmental conditions. Monitoring soil quality and adapting soil conditioning to improve crop yield is one application. Another is tracing and identifying contamination, such as in river water, since our classification model could accurately differentiate between *Aquatic:River* ecosystems and between clean and contaminated ecosystems (i.e., *Mine Effluent*). Finally, a timely application involves the productivity of marine diatoms, currently responsible for up to 20% of global CO_2_ fixation, whose future relies on our ability to monitor diel oscillation. Changes in diatom diversity due to CO_2_-induced ocean acidification could be monitored using either CO_2_ or pH-responsive marine HK sensors as bioindicators (60). In the *Engineered* ecosystems, new sensors can be designed that allow microbes to be triggered appropriately. One attractive application is in a bioreactor, as is maintaining metabolic balance in consortium biomanufacturing such as for anaerobic digestion. Another bioengineering application for HKs is to acclimate a new “wild” strain to industrial fermentation through the introduction of non-native sensors.

The selection of HK sensory domains has allowed us to directly interpret environments in the context of what stimuli organisms that inhabit those environments respond to. We propose that our selection was also fortuitous in terms of ecosystem label predictive capability. Gene content in certain taxa can be highly variable, and many genes carried in a genome are not specifically necessary for survival or operation in a given environment. Prior work suggests that most gene families are extremely rare or unique after analyzing the gene distribution and UniGene richness of clustered genomes (29) and that most bacterial species that have inhabited the Earth are extinct (61). Therefore, species composition, taxonomy, and arbitrary gene function may not be exceptionally predictive of the environment in which they are found. Instead, by focusing on gene classes expected to be enriched in functions necessary for survival, such as sensors, we are more likely to obtain predictive features. Although the inclusion of additional features such as taxonomy or sequence domains might lead to improved model performance, the inclusion of additional features would decrease the model interpretability and lead to a competitive selection of sensory domain compared to other features. We demonstrate that feature importance from classification models is a convenient tool to determine the most impactful sensors in ecosystems or disease states, and we believe this methodology can be applied to similar research especially when a model is built using features with biological interpretability. Our results indicate the microbial sensor profile has the potential to be applied to an array of tasks from ecosystem adaptation and management, medicinal diagnostics, and pharmaceutical targets, for selecting and designing new biosensors in the industry. Since 98.7% of the HKs we use in our analysis are uncharacterized, this can cause difficulties in initial interpretability; however, feature importance allows us to prioritize HKs that are most predictive and discriminatory among different environments. These prioritized sensing domains are critical targets for more in-depth characterization to understand the importance of the signals they are sensing. This work provides a microbial biosensor resource to the scientific community and explores practical applications of sensory domains in future research.

## MATERIALS AND METHODS

### Identification and extraction of HK sensory domains from IMG using Pfam domains

Our focus for this research was on HKs, the most abundant signaling protein in prokaryotes, and their sensory domains. The Pfam database (62), the best-in-class collection of protein families represented using hidden Markov models, was used to identify relevant proteins and their annotations. Specifically, we downloaded all recorded HK proteins from the Interpro website (63, 64) by custom query for the HK IPR conserved domains (IPR003594, IPR003661). The IPR/Pfam annotation names for every sensory domain contained in these HK proteins were extracted, and we added descriptive features for each domain *via* a literature search (Table S1).

The Integrated Microbial Genomes & Microecosystems system (IMG/M) (65) contains an immense wealth of metagenomic data, pulled from sources such as the Department of Energy’s Joint Genome Institute, external scientific studies, and The National Center for Biotechnology Information (NCBI) public sequence archives (65). IMG metagenomes are linked to the Genomes Online Database (GOLD) environmental classification framework (66), which provides ecosystem labels for three classes: *Host-Associated* (e.g., *Animal*), *Engineered* (e.g., *Bioreactor*), and *Environmental* (e.g., *Aquatic, Terrestrial*). From IMG, we obtained all public metagenomes with accompanying Pfam domain annotations (66, 67). Of these, we extracted metagenomes with HK Pfam annotated proteins, leading to 20,712 metagenomes spanning 76 GOLD ecosystems. To estimate sensor abundance, we collected all metagenome gene copy number estimates based on read mapping and enumeration performed by IMG. We filtered to proteins containing both conserved HK Pfam domains (PF02518, PF00512) and cleaved out the sensory domain(s) (Fig. 1). All sensory domains were protein-coding genes. We also identified the associated study name and disease state by cross-referencing GOLD Biosample IDs with NCBI/ENA Biosample IDs provided on the IMG/M web portal (66). A flowchart (Fig. S9) of this process and all metagenomic tables pulled from IMG have been published online for easy access in future work (See Data Availability).

### MMseqs2 clustering of HK sensory proteins

MMseqs2 (Many-against-Many searching) (68) is a software to cluster huge sequence sets into groups of similar sequences, reaching the same sensitivity as PSI-BLAST (37) but magnitudes faster. It is used to maintain key databases like UniProtKB (69). We compared the clusters of full-length HK proteins to individual (extracted) sensory domains but found clustering of full-length proteins led to a dramatic increase in the number of final protein clusters, impacting computational analysis feasibility (Table S2). For this and biologically motivated reasons, we clustered extracted sensory domains only. We also varied MMseqs2 parameters for coverage mode and fraction to obtain the best clusters for analysis (Table S2), along with MMseqs2 methods for taxonomic profiling and protein annotation outside of PSI-BLAST (Fig. S5). In the end, we clustered 21,984,304 proteins with 17,703,911 unique sensory domain sequences, leading to 113,186 clusters. From these, we obtained ~226,000 annotations with 1842 accessions, which after manual data cleaning led to 310 well-characterized HK proteins.

### Histidine kinase annotation with BLASTp and MMseqs2

Annotation of the histidine kinase sensors was desired to assist analysis of the data and focus our attention on specific clusters. To annotate histidine kinase present in metagenomes, we used the MMseqs2 search function to map sensory sequences to Uniprot IDs, using the Uniref50 database (69) and the full-length HK protein sequence. We created a custom script to query Uniprot for name, description, and other identifiers from the MMseqs2 provided Uniprot IDs. From an initial list of proteins, we obtained ~226,000 annotations with 1842 accessions, which after manual data cleaning led to 310 well-characterized HK proteins. Finally, BLASTp was used to verify search results from MMseqs2 for sequences studied in the final results. Where referenced, BLASTp was used to annotate specific sensory domain amino acid sequences, using the default “nr” parameters (results obtained 06/2022-09/2022).

### Ecosystem and sample richness

To provide a broad view of HK domain cluster diversity across and within ecosystems, we calculate two measures. First, Relative Ecosystem Richness is the total of all unique clusters in a given ecosystem divided by the total unique clusters across all ecosystems. This metric shows how much each ecosystem contributes to the global cluster diversity. Ecosystems with high values contribute more to the observed global diversity. Second, Ecosystem Typical Sample Richness is the average number of unique gene clusters in the samples from a given ecosystem divided by the total unique clusters of that ecosystem. This metric is a rough measure of how representative a typical sample is of the cluster diversity within the ecosystem. Ecosystems with high values capture a larger proportion of the cluster diversity in that ecosystem.

### CatBoost input matrix preparation

Prominent machine learning algorithms include LightGBM (Light Gradient Boosted Machine) (70), XGBoost (eXtreme Gradient Boosting) (71), CatBoost (Category Boosting) (72), TabNet (73), and various packages in the scikit-learn package such as RandomForest (74). Initially, we tested our data set using TabNet and CatBoost. CatBoost uses gradient boosting to build decision trees, and it is considered a faster and slightly more accurate method than similar packages LightGBM and XGBoost (75–77). We found CatBoost improved greatly upon TabNet in initial testing (data not shown) and we proceeded to use this package exclusively since a comparison between different machine learning tools has been performed elsewhere and was not the focus of this work. In this work, CatBoost was used for three separate analyses in both regression and classification tasks. First, we built a regression model with the *Aquatic:Marine* ecosystem that could predict physical parameters from the sensory profile (Fig. S3). Next, we built a classifier that could predict the ecosystem a metagenome was drawn from based on its sensory profile, using the GOLD classification ecosystem labels. Finally, we built a classifier in the *Human:Large Intestine* ecosystem that could predict the condition of patients from their sensory profile.

To construct the model training matrix, we selected sensor clusters present in at least 100 metagenomes to limit input data sparsity for CatBoost (14,990 final clusters). For the ecosystem-classifier model, to have enough training samples we selected ecosystems with >30 metagenomes (71 GOLD ecosystems). We also removed “Unclassified” ecosystems from training as these were deemed too ambiguous for comprehensive analysis (66 ecosystems). Our final matrix has 14,990 columns for sensor clusters, and 20,712 rows for metagenomes. The matrix values correspond to the metagenomic sensor abundance, calculated by summing the total cluster’s gene copy number, and dividing by a total protein gene copy number (Fig. 1). The analyses underlying Fig. 1 to 3 in this work use the full 113,876 clusters for an accurate picture of the sensor landscape, but we used the filtered 14,990 with CatBoost to limit matrix sparsity and improve model accuracy. For the disease state classifier, we used conditions with >14 metagenomes and all *Human:Large Intestine* metagenomes.

### CatBoost learning methods for ecosystem and disease-state classification tasks

CatBoost version 1.0.6 was used for all models, and scikit-learn (78) was used for data set splitting and to generate the confusion matrix and F1 score. Shapley is a popular feature importance package, using a polynomial time algorithm to compute optimal explanations based on game theory. SHAP v0.41.0 with TreeExplainer was used (35). Models were trained on the National Energy Research Scientific Computing Center (NERSC) Perlmutter GPU using the “MultiClass” loss function and “Accuracy” custom metric. We used grid search, a standard optimization technique, to tune hyperparameters such as learning rate, tree-depth, and L2 leaf regularization. We found feature selection to have a little or negative impact on model accuracy. Additional details about grid search parameters and feature selection methods are provided in Fig. S4/github.

## Data Availability

GOLD identifiers for all studies used in this research and their corresponding ecosystems excel with accessory data for the marine ecosystem and gut disease classes, and all code for data preparation, ML, Spearman coefficient, and analysis ( github; DOI 10.5281/zenodo.7444768). All metagenome annotation data are public and available in IMG. The matrices, amino acid sequence catalog, and initial FASTA files used for MMseqs2 can be found here (DOI 10.5281/zenodo.7412647). For a full description of the files uploaded, please refer to the "description.ipynb" notebook in the permanent Zenodo address. Finally, the input data tables extracted from IMG that were used to isolate the HK proteins can be found *via* the following public directory link ( https://portal.nersc.gov/project/kbase/publications/HK_sensor_2022).

## SUPPLEMENTAL MATERIAL

Fig. S1 msystems.00026-23-s0001.pdf Clusters found in rare ecosystems and ecosystem tree enrichment.

Fig. S2 msystems.00026-23-s0002.pdf Additional labels for ecosystem beta diversity and cluster richness.

Fig. S3 msystems.00026-23-s0003.pdf CatBoost regression model and methods with focus on temperature for feature importance interpretation.

Fig. S4 msystems.00026-23-s0004.pdf Confusion Matrix for ecosystem-classifier and supplemental methods for CatBoost training.

Fig. S5 msystems.00026-23-s0005.pdf Correlation matrix for ecosystems and table of the top most correlated ecosystems.

Fig. S6 msystems.00026-23-s0006.pdf VicK distribution across different ecosystems and methods for MMseqs2 taxonomy profiling and annotation.

Fig. S7 msystems.00026-23-s0007.pdf QseC tSNE, annotations for correlated clusters, and Disease correlation matrix.

Fig. S8 msystems.00026-23-s0008.pdf Flowchart for data preparation from IMG.

Table S1 msystems.00026-23-s0009.pdf Pfam/IPR for all sensory domains and conserved domains used in this study.

Table S2 msystems.00026-23-s0010.pdf MMseqs2 testing and final clustering parameters.

ASM does not own the copyrights to Supplemental Material that may be linked to, or accessed through, an article. The authors have granted ASM a non-exclusive, world-wide license to publish the Supplemental Material files. Please contact the corresponding author directly for reuse.
