# Supplementary material for: A bacterial sensor taxonomy across earth ecosystems for machine learning applications
Source: mSystems. 2023 Dec 11;9(1):e00026-23. doi: 10.1128/msystems.00026-23 (PMC10804942; doi:10.1128/msystems.00026-23)
Supplement: Fig. S1 — Clusters found in rare ecosystems and ecosystem tree enrichment. [file msystems.00026-23-s0001.pdf]

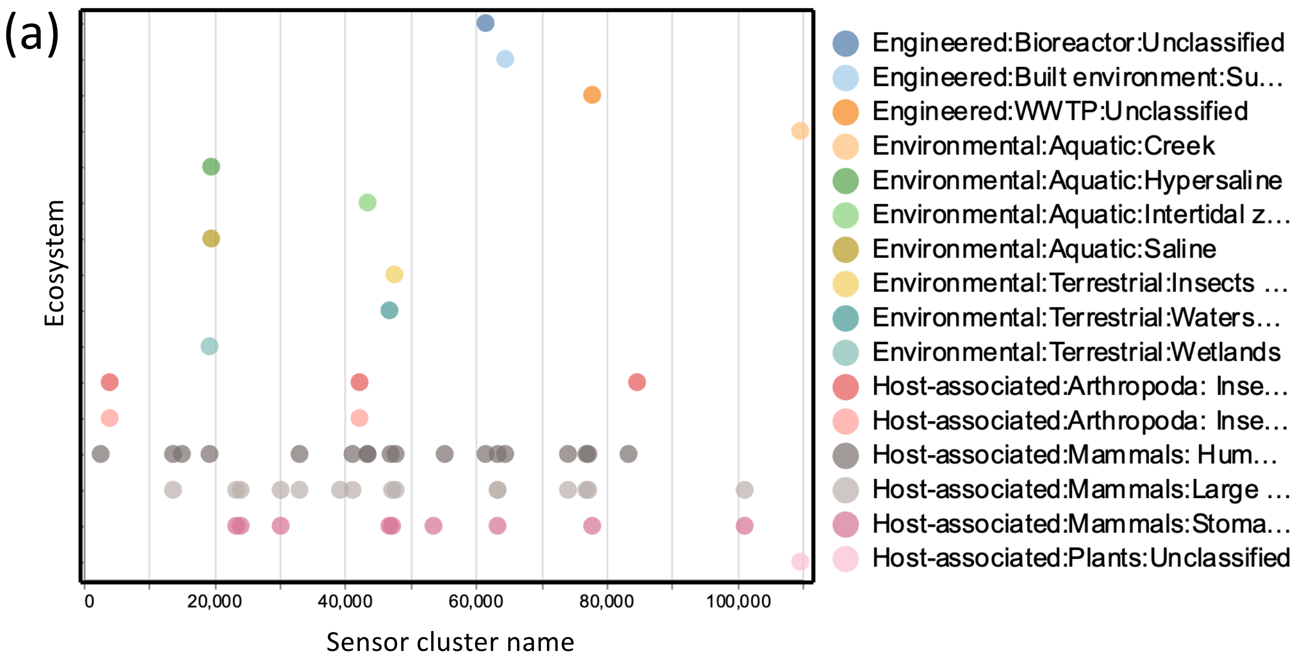


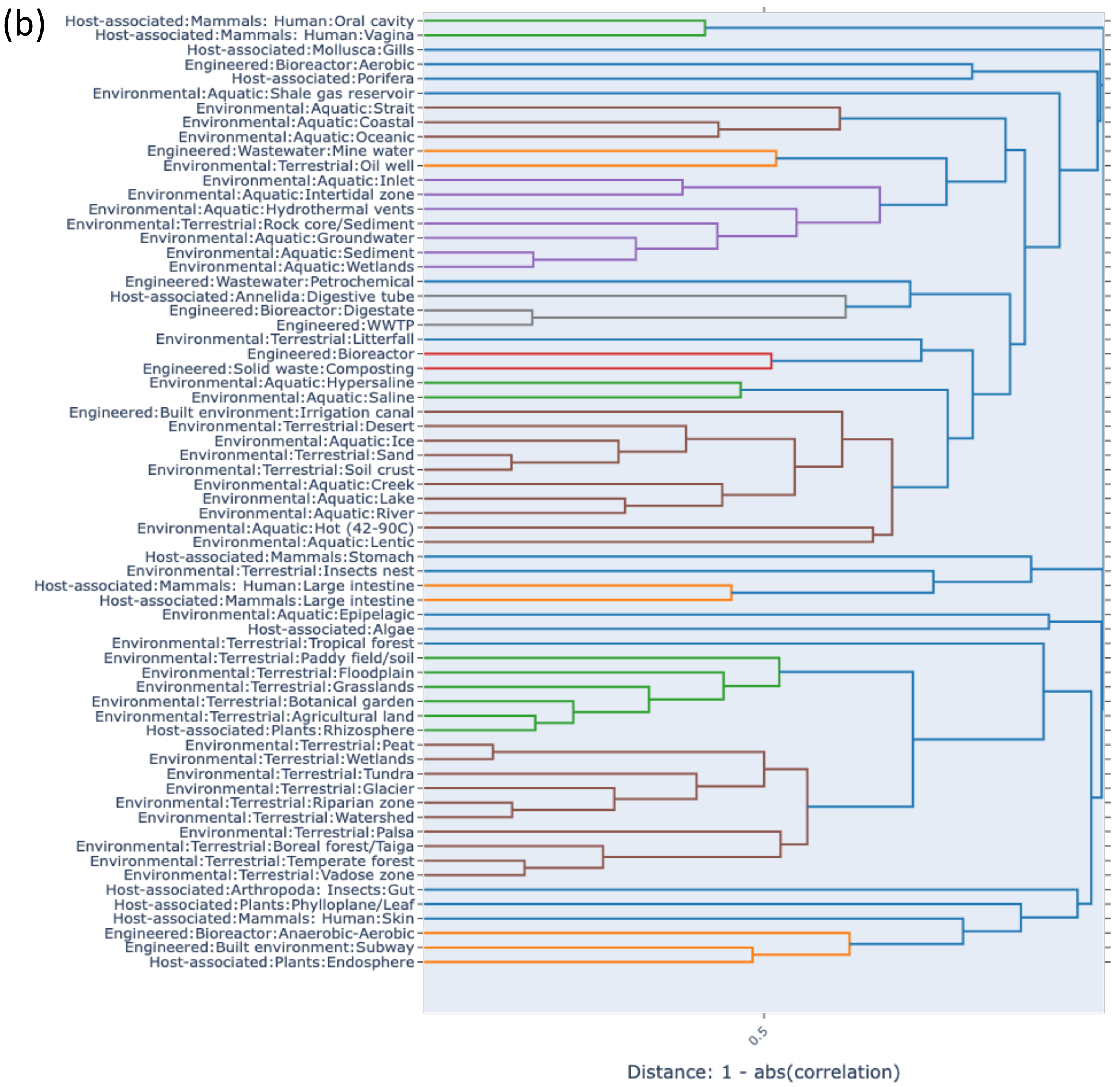


FIG S1: Clusters found in rare ecosystems and ecosystem tree enrichment (a) The sensor protein clusters that are most rare in the sensor profile matrix. Specifically, these clusters are only found in one or two ecosystems. Most rare clusters are from *Human: Large Intestine*, *Mammals: Large Intestine*, and *Mammals: Stomach*, though a few other ecosystems also contain rare clusters. We suggest the *Human:Large Intestine* has the highest number of rare clusters, the highest fraction of sensory proteins, but a relatively low sensor diversity compared to other ecosystems. (b) Tree enrichment diagram using hierarchical clustering for ecosystems using their sensor profile, to supplement **FIG 2**. Similar ecosystems tend to cluster together as indicated by the colored tree branches, indicating there is a predictable structure in the cluster profile between ecosystems.
