# Supplementary material for: A bacterial sensor taxonomy across earth ecosystems for machine learning applications
Source: mSystems. 2023 Dec 11;9(1):e00026-23. doi: 10.1128/msystems.00026-23 (PMC10804942; doi:10.1128/msystems.00026-23)
Supplement: Fig. S2 — Additional labels for ecosystem beta diversity and cluster richness. [file msystems.00026-23-s0002.pdf]

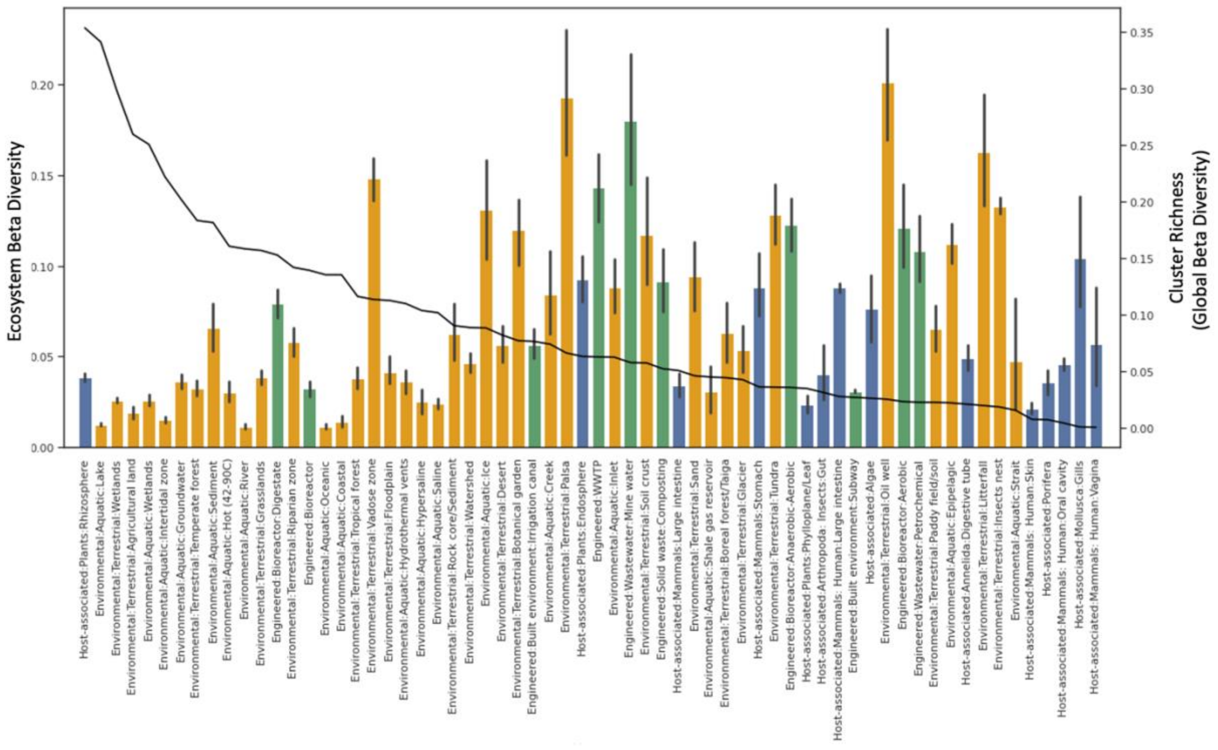


FIG S2: Additional labels for ecosystem beta diversity and cluster richness

Supplementary to **FIG 3a** beta diversity plot, to provide labels for all plot points. The ecosystems are ranked left to right by cluster richness (black line.) The bars correspond to the ecosystem beta diversity. We found that the ecosystems *Wastewater:Mine water* and *Terrestrial:Oil well* had high ecosystem beta diversity, in these ecosystems it appears the diversity of sensors is not as narrow as other ecosystems. Others, like *Human: Large Intestine* and *Aquatic: Lake, Aquatic: Ocean* have small ecosystem beta diversity, indicating sensor profiles are more consistent across all studies in the dataset.
