# Supplementary material for: A bacterial sensor taxonomy across earth ecosystems for machine learning applications
Source: mSystems. 2023 Dec 11;9(1):e00026-23. doi: 10.1128/msystems.00026-23 (PMC10804942; doi:10.1128/msystems.00026-23)
Supplement: Fig. S3 — CatBoost regression model and methods with focus on temperature for feature importance interpretation. [file msystems.00026-23-s0003.pdf]

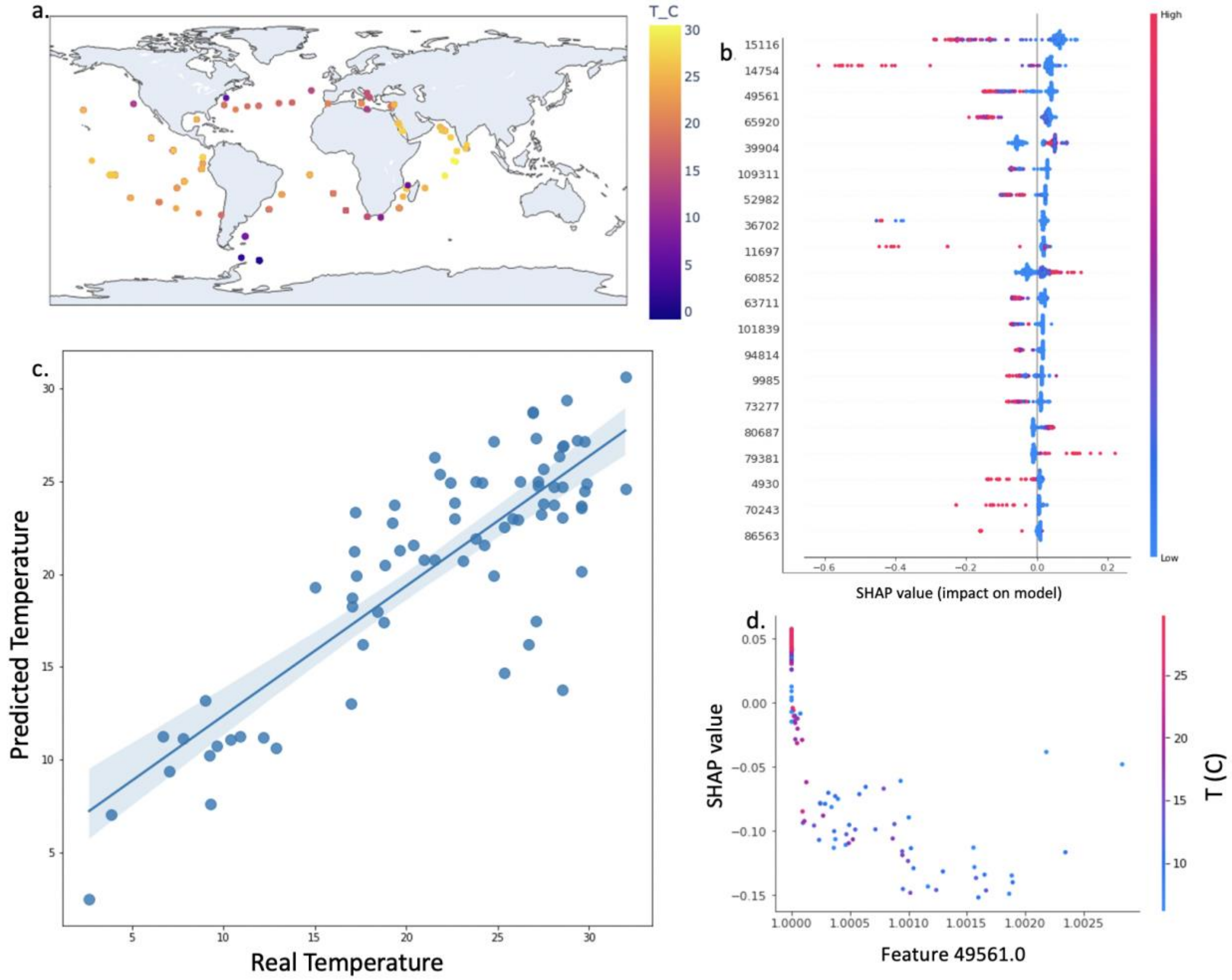

Metric	R2 score-Train	R2 score - Test	Learning rate	bootstrap	l2 leaf reg
**Temperature**	99.5	82.7	0.1	Bayesian	1
**Salinity***	98.6	83.7	0.01	No	25
**pH**	99.5	83.2	0.1	Bayesian	25
**Nitrogen**	94.7	83.7	0.01	Bernoulli	1
**Oxygen**	98.2	74	0.001	Bayesian	10
**Chlorophyll**	97.9	60.8	0.1	Bayesian	1

FIG S3: CatBoost regression model and methods with focus on Temperature for feature importance interpretation

Table shows the R2 score for test and training sets for each model, and the final model parameters after grid search. Multiple metrics obtained an R2 score above 0.83 (a) World map

showing the locations and magnitudes for temperature values in the marine ecosystem subset. (b) Feature importance for the top 20 most impactful features (sensors) that the model uses to predict temperature values. (c) The predicted temperature values vs. real values from the test dataset. (d) Feature importance profile for sensor ***cluster*:*49561***, which is found in SAR324 hydrothermal plume bacteria.

Supplemental results for CatBoost regression model to predict physical parameters from sensor profile

We built ML models to test regression prediction power using our input sensor matrix. We hoped for these regression models to test if the sensor matrix could accurately predict physical parameters in the dataset. We first searched through IMG study data drawn from our initial data gathering, looking for accessory measurements that had enough metagenomes for a regression model. We extracted and cleaned (e.g. standardizing units) physical parameters for each from IMG study data for the *Environmental:Aquatic:Marine* ecosystem, leaving 165-210 metagenomes depending on the physical measurement. The final regression models had physical parameters for temperature, pH, salinity, chlorophyll, nitrogen, and oxygen content in the *Marine* ecosystem. **FIG S1a** shows the locations of ocean metagenomes corresponding to temperature measurements specifically.

For each physical parameter, a CatBoost regression model was built and optimized using grid search. For all, epochs: 5000, depth=8, loss function: RMSE, time < 30s, regression metric: R2 score. Final R2 score after grid search is shown in **FIG S1 Table**, see also github for methods. Our temperature, pH, and N_2 models obtained 82-84% R2 score, while oxygen and chlorophyll had a lower score. Notably, salinity also obtained a high score; however upon closer inspection its profile did not exhibit a predictable range, since most metagenomes were at typical ocean-water salinity with a few divergent outliers. We deemed the salinity model did not have enough data points for meaningful interpretation.

The temperature regression model had the broadest predictable range and was used for further interpretation. Temperature's prediction vs. ground truth labels are shown in **FIG S1c**. Feature importance can help elucidate which features (sensory-domain clusters) are most important during model training in temperature prediction, and we use the importance to see how a feature impacts the final temperature value. It is worth clarifying that covariance must be taken into account in feature importance interpretation, as feature importance represents a feature's responsibility for a change in the model output. Therefore, the most important features are not necessarily temperature sensing proteins, but could be other sensors that trace stimuli that track with temperature. Nevertheless, we introduce the method for feature importance with sensory domains as a new method to identify novel sensors for important stimuli.

Unsurprisingly, when we pulled the top 20 features (**FIG S1b**), nearly all sensory proteins were not annotated and therefore unknown, though taxonomic classification indicated the HK proteins are found within marine bacteria. Notably, in the top 20 features, ***cluster*:*49561*** is annotated upon BLASTp to be an unknown HK found in SAR324, a marine organism

documented to thrive in hydrothermal plumes (60). For this sensor, high feature importance corresponds to higher temperature (**FIG S1d**). We propose that this sensor would be a good target for exploration and experimentation. Therefore, our results suggest sensor profiles may be appropriate for physical parameter prediction and can act as a guide to prioritize new sensor discovery.

To quickly shed clarity on the feature values and interpretation: the rank for a feature indicates how impactful that feature is in the ecosystem classification, and the features are sorted by the sum of values across all metagenomes in order to understand the distribution of impact a feature has on the output class. In the scatter plot for feature importance, the y-axis is the feature's importance rank, the x-axis is the feature importance value and each dot represents one metagenome. A positive x-axis value will lead the model to select for the class, a negative value to select against the class, and the color spectrum represents the value of the feature compared to other classes (red: high-abundance, blue: low-abundance.)
