# Supplementary material for: A bacterial sensor taxonomy across earth ecosystems for machine learning applications
Source: mSystems. 2023 Dec 11;9(1):e00026-23. doi: 10.1128/msystems.00026-23 (PMC10804942; doi:10.1128/msystems.00026-23)
Supplement: Fig. S4 — Confusion Matrix for ecosystem-classifier and supplemental methods for CatBoost training. [file msystems.00026-23-s0004.pdf]

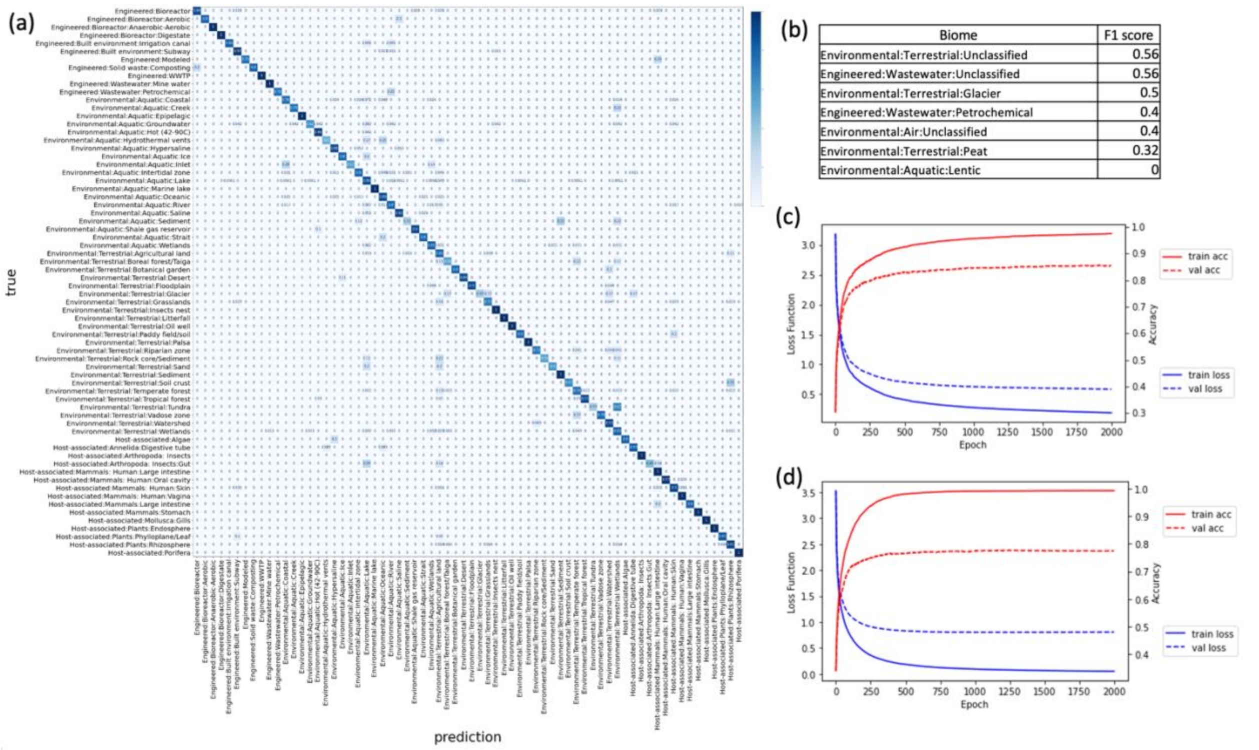


| Biome | F1 score |
|---|---|
| Environmental:Terrestrial:Unclassified | 0.56 |
| Engineered:Wastewater:Unclassified | 0.56 |
| Environmental:Terrestrial:Glacier | 0.5 |
| Engineered:Wastewater:Petrochemical | 0.4 |
| Environmental:Air:Unclassified | 0.4 |
| Environmental:Terrestrial:Peat | 0.32 |
| Environmental:Aquatic:Lentic | 0 |

## FIG S4: Confusion Matrix for ecosystem-classifier and supplemental methods for CatBoost training

(a) The confusion matrix for the final post-grid search CatBoost classifier model. As described in the text, the confusion matrix reveals where the model is mispredicting one ecosystem for another, perhaps revealing which ecosystems are difficult to differentiate. (b) Ecosystems that have the worst performance in the CatBoost classifier. Notably, *Aquatic:Lentic* and *Aquatic:Peat* both performed poorly no matter what parameters we used. We suspect these ecosystems are an amalgamation of similar environmental ecosystems with similar and sometimes synonymous class labels that are inconsistently applied by microbiome investigators. (c, d) Training curve for the CatBoost classifier with (c) MMseqs2 clusters as features and (d) Pfam IDs as features.

## Supplemental Methods for CatBoost classifiers

Additional methods for CatBoost classifiers. For ecosystem classifier, hyperparameters for training were selected using a grid search that was scoped as {*'learning_rate': [0.1, 0.01, 0.3], 'l2_leaf_reg': [5, 10, 25], 'border_count':[5,200], 'bagging_temperature':[0.03,0.25,0.75], 'random_strength':[0.2,0.5,0.8], 'bootstrap_type':['Bayesian', 'Bernoulli', 'No'], 'max_ctr_complexity':[1,5], 'depth': [6, 8, 10]*}. Optimal tuned parameters were {*'l2_leaf_reg': 10, ''border_count': 200, 'max_ctr_complexity': 1, 'depth': 8, 'bagging_temperature': 0.25, 'random_strength': 0.5, 'learning_rate': 0.1, 'bootstrap_type': 'Bayesian'*}. All models were trained for 5000 epochs. Feature selection is one method commonly used to improve model performance by allowing the model to focus on only important features. We tried multiple well-known feature selection methods (1-ANOVA, Recursive Feature Elimination (RFE), and removing low variance and/or correlated features), however, each of these had little or negative impact on model accuracy.

After a grid search for hyperparameter tuning, we obtained 0.87 accuracy in both test and validation sets, and 0.98 for training. Notably, when testing CatBoost with all sensory clusters (113,187) columns we experienced around a 5-fold slowdown (5 min to 25 min) for a training of 5000 epochs and reached an accuracy of 0.815 for test, 0.814 for validation datasets. This indicates the cluster cutoff to limit to 14,990 is acceptable and indeed improves model performance. Broadly speaking, we found *Environmental* ecosystems had the lowest accuracy, for example *Terrestrial:Peat* and *Terrestrial:Lentic* had a F1 score near zero no matter our adjustments (**FIG S4**). We suspect this indicates labeling discrepancy, or perhaps these ecosystem's sensor profiles are indistinguishable from other *Terrestrial* ecosystems.

For the disease state classifier, a hyperparameter grid search was performed on the model using the same parameter range as the ecosystem-classifier; we found the optimal parameters were {*learning_rate:0.1, depth:6, l2_leaf_reg:10, bootstrap_type:'Bernoulli', random_strength:30, iterations:2000*}. We again found hyperparameter tuning had little impact on model performance.
