# Supplementary material for: A bacterial sensor taxonomy across earth ecosystems for machine learning applications
Source: mSystems. 2023 Dec 11;9(1):e00026-23. doi: 10.1128/msystems.00026-23 (PMC10804942; doi:10.1128/msystems.00026-23)
Supplement: Fig. S5 — Correlation matrix for ecosystems and table of the top most correlated ecosystems. [file msystems.00026-23-s0005.pdf]

(a)

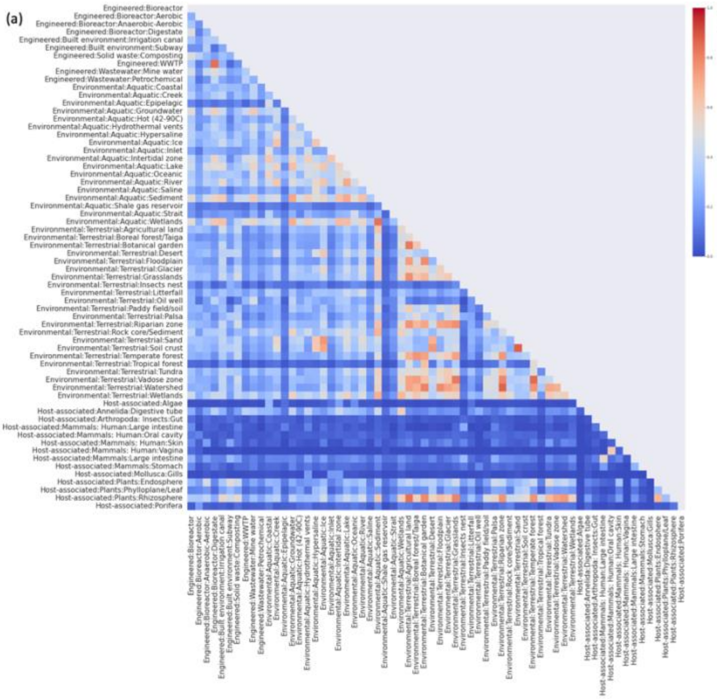

(b)

Ecosystem - 1	Ecosystem - 2	Corr
Environmental:Terrestrial:Sand	Environmental:Terrestrial:Soil crust	0.871640
Environmental:Terrestrial:Soil crust	Environmental:Terrestrial:Sand	0.871640
Environmental:Terrestrial:Watershed	Environmental:Terrestrial:Riparian zone	0.870659
Environmental:Terrestrial:Riparian zone	Environmental:Terrestrial:Watershed	0.870659
Environmental:Terrestrial:Temperate forest	Environmental:Terrestrial:Vadose zone	0.852448
Environmental:Terrestrial:Vadose zone	Terrestrial:Temperate forest	0.852448
Engineered:Bioreactor:Digestate	Engineered:WWTP	0.841116
Engineered:WWTP	Engineered:Bioreactor:Digestate	0.841116
Environmental:Aquatic:Wetlands	Environmental:Aquatic:Sediment	0.839581
Environmental:Aquatic:Sediment	Environmental:Aquatic:Wetlands	0.839581
Environmental:Terrestrial:Agricultural land	Host-associated:Plants:Rhizosphere	0.836663
Host-associated:Plants:Rhizosphere	Terrestrial:Agricultural land	0.836663
Environmental:Terrestrial:Vadose zone	Environmental:Terrestrial:Grasslands	0.819728
Environmental:Terrestrial:Grasslands	Environmental:Terrestrial:Vadose zone	0.819728
Environmental:Terrestrial:Agricultural land	Terrestrial:Botanical garden	0.818205
Environmental:Terrestrial:Botanical garden	Terrestrial:Agricultural land	0.818205
Environmental:Terrestrial:Grasslands	Host-associated:Plants:Rhizosphere	0.798523
Host-associated:Plants:Rhizosphere	Environmental:Terrestrial:Grasslands	0.798523
Environmental:Terrestrial:Watershed	Terrestrial:Botanical garden	0.796926
Environmental:Terrestrial:Botanical garden	Environmental:Terrestrial:Watershed	0.796926
Environmental:Terrestrial:Vadose zone	Environmental:Terrestrial:Riparian zone	0.790528
Environmental:Terrestrial:Riparian zone	Environmental:Terrestrial:Vadose zone	0.790528
Host-associated:Plants:Rhizosphere	Terrestrial:Botanical garden	0.780766
Environmental:Terrestrial:Botanical garden	Host-associated:Plants:Rhizosphere	0.780766
Environmental:Terrestrial:Floodplain	Terrestrial:Botanical garden	0.780213
Environmental:Terrestrial:Botanical garden	Environmental:Terrestrial:Floodplain	0.780213
Environmental:Terrestrial:Floodplain	Environmental:Terrestrial:Watershed	0.777602
Environmental:Terrestrial:Watershed	Environmental:Terrestrial:Floodplain	0.777602
Environmental:Terrestrial:Agricultural land	Environmental:Terrestrial:Grasslands	0.776790
Environmental:Terrestrial:Grasslands	Terrestrial:Agricultural land	0.776790

FIG S5 (a) Correlation matrix for ecosystems, and (b) table of the top most correlated ecosystems.

Both (a,b) indicate some ecosystems are noticeably correlated. We found, interestingly, high correlation does not equate to ML misclassification, while other less correlated ecosystems can be mispredicted.

We recognize there may be higher order interactions between sensors within HK proteins that are not captured in our sensor cluster profile. Further feature engineering efforts could add complexity and improve performance.
