# Supplementary material for: A bacterial sensor taxonomy across earth ecosystems for machine learning applications
Source: mSystems. 2023 Dec 11;9(1):e00026-23. doi: 10.1128/msystems.00026-23 (PMC10804942; doi:10.1128/msystems.00026-23)
Supplement: Fig. S6 — VicK distribution across different ecosystems and methods for MMseqs2 taxonomy profiling and annotation. [file msystems.00026-23-s0006.pdf]

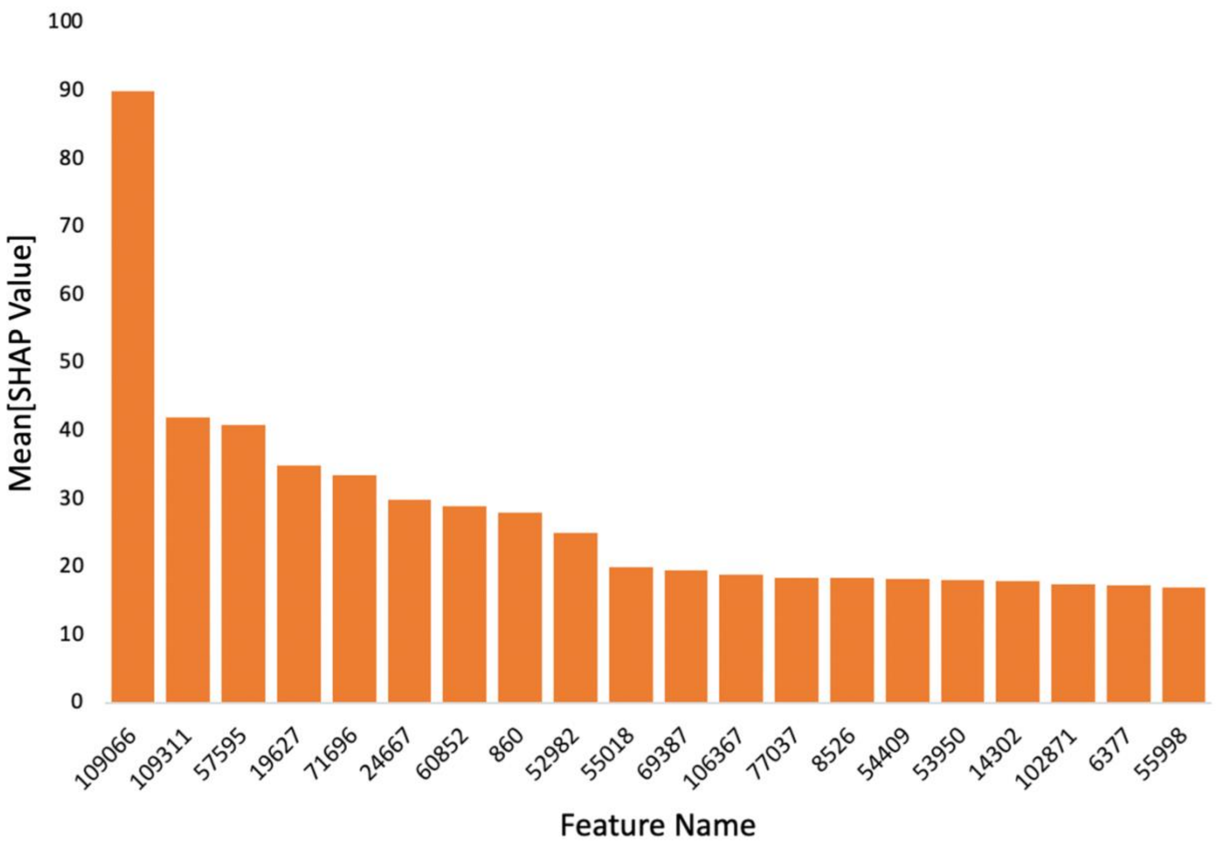


FIG S6: Top Features for the Ecosystem Label Classifier

Top most predictive features for the ecosystem label classifier, and are discriminatory for the prediction of all classes. Each feature stands for a cluster of sensory proteins, and the features are ranked by their absolute SHAP value. A few of the top clusters had annotations in the literature, for example the top features (***cluster:109066***, ***cluster:109311***) *are putative oxygen sensors analogous to fixL/dosP*. Meanwhile, ***cluster:71696*** is annotated to be a KdpD sensor, and is found in every ecosystem in the dataset and ***cluster:860*** is annotated to be FilI, a methanogenesis regulator. ***Cluster:60852*** is annotated to be ntrY, regulating the transcription of a series of virulence factors and capsular polysaccharide production. Finally, ***cluster:69387*** is annotated to be a blue-light activated protein, PS-LOV-histidine kinase, related to or including phytochromes. Each feature can be annotated and explored in further detail, though certain feature clusters do not have literature annotation, or their annotation is not constrained to a single HK-sensor type.
