# Supplementary material for: A bacterial sensor taxonomy across earth ecosystems for machine learning applications
Source: mSystems. 2023 Dec 11;9(1):e00026-23. doi: 10.1128/msystems.00026-23 (PMC10804942; doi:10.1128/msystems.00026-23)
Supplement: Fig. S7 — QseC tSNE, annotations for correlated clusters, and Disease correlation matrix. [file msystems.00026-23-s0007.pdf]

(a)

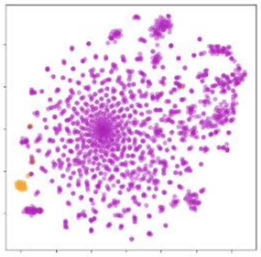

(b)

	Average Abundance				
**Cluster**	**Adult**	**Newborn**	**Name**	**Stimulus / Activation**	**BLASTp organism results**
**112899**	3.03E-04	1.59E-03	arcB	Oxygen; regulates oxidative / fermentative catabolism gene transcription.	Klebsiella pneumoniae, E coli
**10655**	2.14E-04	7.14E-04	atoS	Acetoacetate and spermidine, regulation motility / chemotaxis, flagellar regulon	Escherichia coli, Klebsiella pneumoniae
**96765**	4.33E-04	1.64E-03	citA	Citrate, catabolism of citrate	Ecoli, Klebsiella pneumoniae
**5255**	3.04E-04	1.70E-03	cpxA	KCl, pH, induce biofilm	Escherichia col, Burkholderia pseudomallei, Staphylococcus aureus
**16205**	5.39E-04	2.07E-03	dcuS	C4 (fumarate, succinate, malate...)	Escherichia coli
**15116**	3.91E-05	1.71E-03	dcuS	C4 (fumarate, succinate, malate...)	Escherichia coli
**112837**	5.53E-04	2.77E-03	dcuS	C4 (fumarate, succinate, malate...)	Escherichia coli
**27001**	4.60E-04	2.65E-03	DpiB	Citrate	Escherichia coli
**66664**	4.29E-04	1.99E-03	evgS	Acidic pH, aerobic conditions	Escherichia coli, Shigella sonnei
**21148**	4.27E-04	2.02E-03	evgS	Acidic pH, aerobic conditions	Escherichia coli, Shigella sonnei
**8526**	3.08E-04	1.66E-03	glnL	Glutamate	Enterobacter hormaechei, Serratia marcescens
**55998**	2.66E-04	1.43E-03	phoR	Phosphate absense	Citrobacter freundii
**72684**	3.26E-04	1.13E-03	qseC	Quorum sensing (AI-3, iron)	
**57709**	2.83E-04	1.74E-03	uhpC	Glucose-6-phosphate	Escherichia coli
**79703**	3.41E-04	1.11E-03	ydpa		
**27946**	4.04E-04	1.83E-03	ypdA	Pyruvate	Phytobacter, Grimontella, Enterobacter, Metakosakonia, Citrobacter

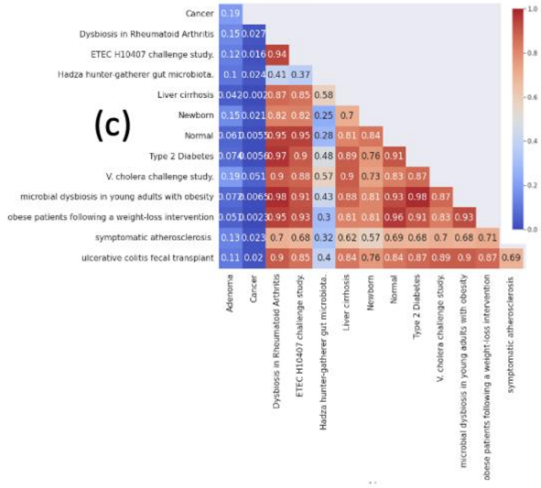

FIG S7: QseC tSNE, annotations for correlated clusters, and Disease correlation matrix

(a) tSNE for all *Human:Large Intestine* sensory domain clusters, QseC cluster in orange color. Upon further investigation, we found the clusters in the QseC grouping are correlated. Literature search indicates prior research on correlated clusters has proposed linkages and modulation of human disease states. QseC and these correlated protein clusters we found were an identifier for the infant gut (**FIG 5**). (b) Brief description of QseC correlated clusters from (a). The second column shows the average abundance for these clusters in infants, the first column in adults. Names and annotations for these sensory domains after BLASTp and literature search are shown in columns 3-5. (c) Correlation matrix for disease states in *Human:Large Intestine*. We notice that adenoma, cancer and Hazda conditions are especially uncorrelated to the rest of the conditions, however most conditions are quite correlated.
