# Supplementary material for: A bacterial sensor taxonomy across earth ecosystems for machine learning applications
Source: mSystems. 2023 Dec 11;9(1):e00026-23. doi: 10.1128/msystems.00026-23 (PMC10804942; doi:10.1128/msystems.00026-23)
Supplement: Fig. S8 — Flowchart for data preparation from IMG. [file msystems.00026-23-s0008.pdf]

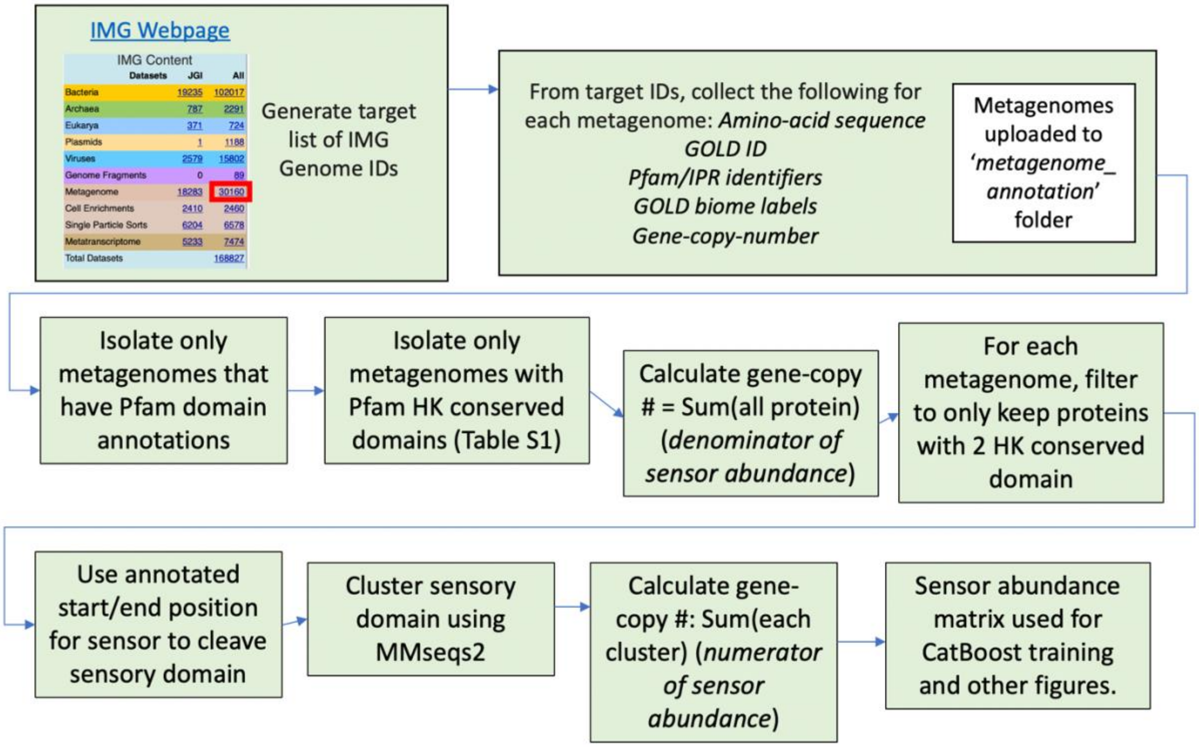

FIG S8: Flowchart for Data Preparation from IMG

Process for initial extraction of IMG Genome IDs. After genome IDs were isolated from the public database, target IDs were used to collect the amino acid sequence, GOLD identifier, PFAM and IPR domains, and other information. Next, only metagenomes that had been annotated with Pfam domains and that contained HK conserved Pfam domains were used for further analysis. The metagenome files that contain conserved HK Pfam domains are published and freely available (see: Data Availability). Notably, metagenomes at this stage contain all proteins including HKs and other proteins. Final CatBoost training matrix used has also been published (see: Data Availability.)
