# Supplementary material for: A bacterial sensor taxonomy across earth ecosystems for machine learning applications
Source: mSystems. 2023 Dec 11;9(1):e00026-23. doi: 10.1128/msystems.00026-23 (PMC10804942; doi:10.1128/msystems.00026-23)
Supplement: Table S1 — Pfam/IPR for all sensory domains and conserved domains used in this study. [file msystems.00026-23-s0009.pdf]

Table S1: Pfam/IPR for all sensory domains and conserved domains used in this study

The Pfam/InterPro ID for two conserved domains used for HK identification are listed along with all sensory domains extracted from each HK on the InterPro website. Name / description for each sensor is filled in the table after literature search and validation.

| Pfam | InterPro | Name | Type | Description |
|---|---|---|---|---|
| **PF02518** | **IPR003594** | **HATPase_c** | **conserved** | Histidine kinase/HSP90-like ATPase |
| **PF00512** | **IPR003661** | **HisKA** | **conserved** | Signal transduction histidine kinase, dimerisation/phosphoacceptor domain |
| PF08521 | IPR013727 | 2CSK_N | sensor | This domain is found in bacterial two-component sensor kinases towards the N terminus. |
| PF07694 | IPR011620 | 5TM-Lyt | sensor | Signal transduction histidine kinase, 5TM receptor LytS, transmembrane region |
| PF07695 | IPR011623 | 7TM-DISM | sensor | 7TM-DISM receptor, extracellular domain, type 1 |
| PF07696 | IPR011622 | 7TM-DISM-2 | sensor | 7TM-DISM receptor, extracellular domain, type 2 |
| PF18719 | IPR041610 | ArlS | sensor | ArlS, sensor domain |
| PF17200 | IPR033480 | Cache_2 | sensor | Cache is an extracellular domain that is predicted to have a role in small-molecule recognition |
| PF03924 | IPR006189 | CHASE | sensor | This domain is found in the extracellular portion of receptor-like proteins such as serine/threonine |
| PF05227 | IPR007891 | CHASE_3 | sensor | CHASE3 is an extracellular sensory domain, which is present in various classes of transmembrane reception |
| PF05226 | IPR007890 | CHASE2 | sensor | CHASE2 is an extracellular sensory domain, which is present in various classes of transmembrane reception |
| PF05228 | IPR007892 | CHASE4 | sensor | CHASE4 |
| PF17150 | IPR033415 | CHASE6_C | sensor | C-terminal domain of two-partite extracellular sensor domain |
| PF17152 | IPR033417 | CHASE8 | sensor | Periplasmic sensor domain CHASE8 |
| PF16527 | IPR032404 | CpxA_peri | sensor | Two-component sensor protein CpxA, periplasmic domain |

| | | | | |
|---|---|---|---|---|
| PF02743 | IPR033479 | dCache_1 | sensor | Double Cache domain 1 |
| PF13185,PF13492,PF01590 | IPR003018 | GAF | sensor | Domain present in phytochromes and cGMP-specific phosphodiesterases. |
| PF18698 | IPR041328 | HisK_sensor | sensor | ResE, histidine kinase sensor domain |
| PF16927 | IPR031621 | HisKA_7TM | sensor | Histidine kinase, N-terminal 7TM region |
| PF09385 | IPR018984 | Histidine_kinase_N | sensor | Histidine kinase, N-terminal |
| PF16750 | IPR031930 | HK_sensor | sensor | Two-component histidine kinase, sensor domain |
| PF02702 | IPR003852 | KdpD | sensor | Signal transduction histidine kinase, osmosensitive K+ channel sensor, N-terminal |
| PF13493 | IPR025201 | KdpD transmembrane | sensor | Sensor protein KdpD, transmembrane domain |
| PF16767 | IPR031909 | KinB_sensor_dom | sensor | Alginate biosynthesis sensor protein KinB, sensor domain |
| PF05231 | IPR007895 | MASE1 | sensor | 8-TMR integral-membrane domain, with conserved residues (3 Pro, 3 Trp intrahelical); stimuli unknown |
| PF00015 | IPR004089 | MCP | sensor | TM sensor proteins consisting of a periplasmic input domain (CACHE, PAS, GAF, or TarH) a |
| PF03707 | IPR005330 | MHYT | sensor | 6 TMR with short linkers; characteristic intrahelical MH(YT) motif in TMR2, TMR4, and TMR6 |
| PF13188, PF13426 | IPR000014 | PAS | sensor | PAS domain; PAS motifs appear in archaea, eubacteria and eukarya. |
| PF08446 | IPR013654 | PAS-2 | sensor | Pas domain; PAS motifs appear in archaea, eubacteria and eukarya. |
| PF08447 | IPR013655 | PAS-3 | sensor | Pas domain; PAS motifs appear in archaea, eubacteria and eukarya. |
| PF08448 | IPR013656 | PAS-4 | sensor | Pas domain; PAS motifs appear in archaea, eubacteria and eukarya. |
| PF00989 | IPR013767 | PAS-fold | sensor | Pas-fold; PAS motifs appear in archaea, eubacteria and eukarya. |
| PF00497 | IPR001638 | PBPb | sensor | Bacterial periplasmic substrate-binding proteins bacterial proteins, eukaryotic ones are in P |
| PF00060 | IPR001320 | PBPe | sensor | Eukaryotic homologues of bacterial periplasmic substrate binding proteins. Prokaryotic homologue |

| | | | | |
|---|---|---|---|---|
| PF16736 | IPR031967 | PhoR_single_Cache-like_dom | sensor | PhoR, single Cache-like domain |
| N/A | IPR044767 | Phytochrome | sensor | Phytochrome A/B/C/D/E-like, histidine-kinase-related domain |
| PF00360 | IPR013515 | Phytochrome_cen-reg | sensor | Phytochrome, central region |
| PF07494 | IPR011110 | Reg_prop | sensor | 14 tandem repeats of 14 aa each form 2 7-bladed β-propellers |
| PF16524 | IPR032408 | RisS_PPD | sensor | This entry represents the periplasmic domain of the sensor histidine kinases such as RisS. |
| PF17203, PF17202 | IPR033463 | sCache_3 | sensor | Single cache domain 3 |
| PF13755 | IPR025908 | Sensor_TM1 | sensor | Sensor N-terminal transmembrane domain |
| PF00474 | IPR001734 | SSF | sensor | 13 TMR; catalyze the uptake of a wide variety of solutes (including sugars, proline, and iodide) |
| PF13756 | IPR025919 | Stimulus_sens_dom | sensor | This domain is found in the periplasmic region of the sensor component of the two-component regulator |
| PF02203 | IPR003122 | TarH | sensor | Homologues of the ligand binding domain of Tar |
| PF00582 | IPR006016 | UspA | sensor | This entry represents a domain found in the universal stress protein UspA |
| PF17149 | IPR033414 | CHASE5 | sensor | Periplasmic sensor domain |
