# Supplementary material for: A bacterial sensor taxonomy across earth ecosystems for machine learning applications
Source: mSystems. 2023 Dec 11;9(1):e00026-23. doi: 10.1128/msystems.00026-23 (PMC10804942; doi:10.1128/msystems.00026-23)
Supplement: Table S2 — MMseqs2 testing and final clustering parameters. [file msystems.00026-23-s0010.pdf]

Table S2: MMseqs2 testing and final clustering parameters

Initial testing for MMseqs2 benchmarked with only *Host-associated* ecosystems. Percent coverage, coverage mode, and fasta type (full length or just extracted sensor domain) were varied. Final cluster parameters were selected by targeting a high percent Pfam identity in each cluster, because we expect resulting sequences to roughly encompass a similar function. We used final parameters (coverage mode: 0; coverage: 0.8) that led to the highest percent Pfam identity (81%) of any tested parameter set. After filtering to clusters that are contained in > 100 metagenomes, the number of clusters is 14,990 (see CatBoost data preparation.)

Initial testing for MMseqs2 using only Host-associated ecosystems.

Clustered	# Proteins	# of clusters	% coverage	Coverage mode	% Pfam identity
**Sensor**	**3,888,462**	**33,825**	**0.8**	**0**	**0.812**
Sensor	3,888,462	22,811	0.7	0	0.795
Sensor	3,888,462	84,380	0.6	2	0.772
Sensor	3,888,462	25,516	0.8	3	0.782
Full HK	3,229,635	131,208	0.8	0	0.707

Final parameters for MMseqs2 of sensory proteins in all ecosystems.

Clustered	# Proteins	# of clusters	% coverage	Coverage mode	% Pfam identity
Sensor	21,984,304	113,186	0.8	0	0.803

Supplemental methods for MMseqs2 parameter testing

We varied MMseqs2 parameters for coverage mode and fraction in order to obtain the best parameters for final clustering. The default MMseqs2 parameters are -c (sensitivity) of 0.8, coverage mode (--cov-mod) of 0. Coverage mode of 0 is a bidirectional coverage, where only sequences are clustered that have a sequence length overlap greater than -c% of the longer of the two sequences. We also tried coverage mode 2 and 3, which are query coverage modes in which one alignment is paired against a query. Initial testing was performed on just *Host-associated* ecosystems to limit computer time. Multiple modes [0, 1, 3] and coverage fractions (0.7, 0.8) were tested, and both full-length HK proteins and isolated sensory domains were used for MMseqs2. We determined the best parameters for the final clustering both by quantifying the percent Pfam identity in each cluster, targeting a higher percent Pfam identity because we expect the resulting sequences to roughly encompass a similar function. Upon further investigation, Pfam domains that were not identical in a cluster usually were annotated as "PAS-fold," "PAS-2," "PAS-3," etc., indicating the current popular PAS naming scheme may be incongruent with results obtainable by large-scale protein similarity clustering.
